# The ‘Postural Rhythm’ of the Ground Reaction Force during Upright Stance and Its Conversion to Body Sway—The Effect of Vision, Support Surface and Adaptation to Repeated Trials

**DOI:** 10.3390/brainsci13070978

**Published:** 2023-06-21

**Authors:** Stefania Sozzi, Shashank Ghai, Marco Schieppati

**Affiliations:** 1 Independent Researcher, 27100 Pavia, Italy; sozzi_stefania@hotmail.it; 2 Department of Political, Historical, Religious and Cultural Studies, Karlstad University, 65188 Karlstad, Sweden; shashank.ghai@kau.se; 3 Centre for Societal Risk Research, Karlstad University, 65188 Karlstad, Sweden; 4 Independent Researcher, 20123 Milano, Italy

**Keywords:** human stance, ground reaction force, body sway, spectral analysis, frequency, vision, support surface, adaptation

## Abstract

The ground reaction force (GRF) recorded by a platform when a person stands upright lies at the interface between the neural networks controlling stance and the body sway deduced from centre of pressure (CoP) displacement. It can be decomposed into vertical (VGRF) and horizontal (HGRF) vectors. Few studies have addressed the modulation of the GRFs by the sensory conditions and their relationship with body sway. We reconsidered the features of the GRFs oscillations in healthy young subjects (n = 24) standing for 90 s, with the aim of characterising the possible effects of vision, support surface and adaptation to repeated trials, and the correspondence between HGRF and CoP time-series. We compared the frequency spectra of these variables with eyes open or closed on solid support surface (EOS, ECS) and on foam (EOF, ECF). All stance trials were repeated in a sequence of eight. Conditions were randomised across different days. The oscillations of the VGRF, HGRF and CoP differed between each other, as per the dominant frequency of their spectra (around 4 Hz, 0.8 Hz and <0.4 Hz, respectively) featuring a low-pass filter effect from VGRF to HGRF to CoP. GRF frequencies hardly changed as a function of the experimental conditions, including adaptation. CoP frequencies diminished to <0.2 Hz when vision was available on hard support surface. Amplitudes of both GRFs and CoP oscillations decreased in the order ECF > EOF > ECS ≈ EOS. Adaptation had no effect except in ECF condition. Specific rhythms of the GRFs do not transfer to the CoP frequency, whereas the magnitude of the forces acting on the ground ultimately determines body sway. The discrepancies in the time-series of the HGRF and CoP oscillations confirm that the body’s oscillation mode cannot be dictated by the inverted pendulum model in any experimental conditions. The findings emphasise the robustness of the VGRF “postural rhythm” and its correspondence with the cortical theta rhythm, shed new insight on current principles of balance control and on understanding of upright stance in healthy and elderly people as well as on injury prevention and rehabilitation.

## 1. Introduction

The muscles that keep us upright never rest. Our bipedal stance is strictly controlled by continuous and carefully adjusted neural commands acting onto several muscles with different locations in the body, dissimilar anatomy and primary function, short or long tendons, manifold mechanical effects and contractile characteristics, activated in a tonic or phasic mode [1,2,3,4]. An extremely elaborate neural network [5] must control the level of force in these muscles, able to keep upright the unstable standing body for prolonged periods. At the same time, this network can suddenly switch to a different activation mode when an external event puts the stance at risk [6,7,8,9,10].

It is no wonder, then, that stance control has been addressed by countless investigations, each of which have provided one or more pieces to the puzzle (see for recent reviews, [11,12,13,14]). Most studies have leveraged geometric measures of body sway as an index of stability, recorded by the force platform upon which subjects stand, such as the length of the path travelled by the centre of foot pressure (CoP) or the area of the surface covered by the CoP wandering or related measures [15,16,17]. CoP excursions along the antero-posterior (AP) or medio-lateral (ML) directions of the horizontal plane have served as the basis for indirectly assuming fundamentals of balance control [18,19,20], thereby not implying that the least possible sway corresponds to the highest stability [21,22,23,24].

Few papers have devoted attention to the modulation of the components of the ground reaction forces (GRFs) while standing [25,26,27], probably because many researchers refrained from considering the force platform as an expensive digital weighing-scale. Some of those who did address this issue measured the amplitude of the oscillations of the vertical component of the GRF (VGRF) and the frequency of its oscillations [28,29,30,31,32,33]. Their findings have been matched to the resonant properties of the body [34,35,36] and compared to the coherence of the coordination of the force vectors between the lower limbs [37].

We were among those with an interest in the distinctive attributes of the GRFs because their modulation is at the interface between the neural network(s) controlling upright stance and the traditional geometric measures of body sway, normally considered a proxy for safe standing. In a recent article [33], we reported that the *amplitude* of the VGRF oscillations broadly changed depending on the sensory conditions at hand, e.g., it increased without vision or when standing on a compliant support or both. However, the *frequency* of the continuous VGRF oscillations appeared to be fairly constant with eyes open (EO) or closed (EC) and both on compliant and on hard support, such that the existence of a central “postural rhythm” was posited.

Under both hard and compliant support conditions, the VGRF oscillation frequency was restrained in a common range, even if it appeared to be somewhat higher and less regular on the hard than compliant support. The power spectrum was characterised by a distinct frequency peak around 4–5 Hz, as others had shown before [2,28,29,30,32,38]. Vision and hard support strongly reduced the amplitude of the VGRF oscillations, compared to EC and compliant support, but scarcely affected their dominant frequency. Hence, we agree that a basic rhythm exists in the central nervous system, which materialises on assuming the upright posture and increases in amplitude with higher task demands [39]. We also posit that this fundamental rhythm originates in the brain [see, 5] and is modulated more in amplitude than in frequency by the sensory inflow (the somaesthetic input from the support surface and, possibly, vision) [10,33].

Of note—and remarkably so—the profile of the VGRF power spectrum (its peak frequency and its range of variation from about 2 to 7 Hz) is completely distinct from that of the CoP excursions, where most of the spectrum is contained below 2 Hz regardless of the support surface characteristics, vision or prevalent direction of the body oscillations (AP or ML axis) [32,33]. These observations suggest that the analysis of the CoP, albeit representative of the geometrical sway, gives an incomplete, possibly inadequate insight into the mechanisms controlling upright balance. Hence, we hypothesized that deeper knowledge of the GRF pattern of oscillation would allow gaining insight into the neural mechanisms responsible for balance control and help dissecting out the central from the peripheral influences on sway features.

The oscillations of the vertical component of the GRF are evidence of the vertically aligned motion of the Centre of Mass (CoM) and of its active control. However, the CoM motion does not only contain vertical accelerations. It is characterised by horizontal and rotational accelerations as well [40]. Therefore, a second aim of this study was to describe the oscillations (amplitude and frequency) of the horizontal components of the GRF (HGRFs), and to consider whether and how the changes in the VGRF and HGRF are converted to body sway (the CoP path and frequency of oscillation). In order to enhance variability and challenge the sensory modulation of the neural control, we have modified the visual condition and exploited both hard and compliant supports; we hypothesised that the basic postural rhythm would be minimally affected by the unsecure support surface despite changes in sway amplitude.

In addition, since we have recently shown that short-term adaptation to standing trials repeated in rapid succession had a sizeable influence on some CoP excursion variables [10], we asked whether the fundamental GRF rhythms are modified in the adapted trials. Investigating the effects of adaptation on the amplitude and frequency of the GRF components concurrently with those of the body sway would potentially open an additional window on the factors contributing to the postural rhythm. This approach would allow contrasting sensory and central modulation of balance processes as well, since adaptation is considered a brain process dependent on an internal model [41].

Therefore, we re-considered the features of the GRF oscillations in a population of healthy young subjects, with the aim of characterising the effects of vision, support surface and adaptation on the VGRF and HGRF features. Then, we set out to quantify the degree of modulation of the VGRF and HGRF induced by sensory states and by the central “set” (assumed to differ between the non-adapted and adapted states), as evidence of the afferent and central modulations of the basic rhythm and of its conversion to body sway. The parallel analysis of the GRF oscillations and the CoP motion in the medio-lateral and antero-posterior directions confirmed substantial differences between the GRFs and the geometric measures of sway and emphasised the importance of the frequency analysis of these variables [10,20]. We also quickly compared our data to the available literature and established that this postural VGRF rhythm can be found in elders and patients, adding to the general relevance of this metric.

## 2. Methods

### 2.1. Participants and Procedures

A total of 24 young, healthy subjects (12 females and 12 males) voluntarily participated in this study, which was approved by the local review board (Istituti Clinici Scientifici Maugeri IRCCS, approval number #2564-CE), after signing an informed consent according to the Declaration of Helsinki principles. Their mean age, height and weight (±SD) were 28.6 ± 4.3 years old, 172.0 ± 6.04 cm and 65.6 ± 11.5 kg, respectively. Participants were recruited from resident physicians or physiotherapists at the Istituti Clinici Scientifici Maugeri SB. Participants had no history of neurological or musculoskeletal disorders and had no sight problems, or their visual acuity was corrected. None of the subjects reported major injuries or falls, or ankle sprains. The analysis was performed on previously recorded data [10,33] and on new data recorded from one additional volunteer. Out of all the recruited subjects (n = 26), 2 were excluded from the analysis; 1 voluntarily oscillated as spontaneously declared after several trials, 1 had a smaller number of requested trials, and none of these were available for completing the recording procedure.

Subjects were asked to maintain upright stance for 100 s on a force platform (Kistler 9286BA,Winterthur, Switzerland) with bare feet parallel to each other at hip-width apart in different visual (eyes closed, EC or eyes open, EO) and base of support (solid, S or foam, F) conditions. This resulted in a total of four experimental conditions: eyes closed or eyes open with foam base of support (ECF and EOF) and eyes closed or eyes open with solid base of support (ECS and EOS). The solid support was the force platform, while the foam support was a foam pad (Airex Balance Pad, Sins, Switzerland; L 50 cm, W 41 cm, H 6 cm, density 55 g/dm^3^, Young’s module 260 kPa) placed onto the platform [42].

The outline of the feet was marked on a paper sheet fixed on the top of the platform or on the foam pad, for consistency across consecutive trials. No subjects had ever tried to maintain upright stance on a foam base of support previously. Subjects were not required to stand as still as possible, but stood at ease [23,43] and looked at the structured visual scene of the laboratory wall at 6 m distance [20,44] during the EO trials. Subjects were asked to avoid deliberate movements of the upper body and head (in pitch, roll and yaw). The last 90 s epoch of each 100 s stance trial was acquired [45,46,47] to exclude the adjusting phase occurring after stepping onto the platform or on the foam pad. None of the subjects lost balance, lifted a foot or made a step during the trials. Each experimental session was performed on separate days and the conditions were randomised across subjects. During each session, subjects performed 8 consecutive trials in the same experimental condition, separated by a brief period of rest (less than 60 s). Only the first (non-adapted) and the eighth (adapted) trial of each condition were considered in the subsequent analyses.

### 2.2. Data Acquisition and Analysis

The ground reaction force in vertical (VGRF), antero-posterior (AP HGRF) and medio-lateral (ML HGRF) directions obtained by the force platform and the instantaneous position of the centre of pressure (CoP) both in ML and in AP directions were acquired at a sampling frequency of 140 Hz [38] by the SMART-D Capture software (BTS, Garbagnate Milanese, Italy). All these signals were high-pass filtered at 0.01 Hz and low-pass filtered at 10 Hz with a 4th order Butterworth filter after removing the respective mean values, with a software developed in LabVIEW (National Instruments, Austin, TX, USA).

### 2.3. Frequency–Domain Analysis

We performed the frequency analysis of the platform data (VGRF, HGRF, and CoP position) by means of the auto power spectrum virtual instrument (VI) algorithm of the LabVIEW signal processing functions. This VI calculated the fast Fourier transform of the time-series data of each trial, subject, visual and support condition and produced a single-sided power spectrum (the positive half of the frequency spectrum from 0.01 Hz to 70 Hz). The resolution (sample frequency/sample number of the GRFs and CoP signals) was 0.011 Hz for the sampling frequency of 140 Hz [20,48] and the total sample number over 90 s was 12,600 (=90 * 140).

The power spectrum profile of the VGRF had the shape of a normal distribution curve [33]. For each participant and condition, we fitted the VGRF spectrum with a Gaussian distribution function, $y\left(f\right)=Ae^{-\frac{{\left(f-\mu \right)}^{2}}{2\sigma^{2}}}$, by means of the “Curve Fitting” analysis tool of the software Origin (OriginLab Corp, Northampton, MA, USA), where A is the peak value of the curve, *e* is the Euler number, *µ* represents the frequency (*f*) at which the peak in the power spectrum profile occurs (hereafter, the dominant frequency) and *σ* is the Standard Deviation (SD) of the Gaussian function. For each power spectrum profile, the goodness of fit was estimated by the Pearson’s R coefficient.

In order to detect differences between visual conditions, the values of the VGRF dominant frequency, peak amplitude and SD of the Gaussian curves of each subject in the EC condition were plotted against those in the EO condition. Thus, the distribution of the data points obtained were studied by a linear regression model and the R^2^ was calculated. In order to investigate whether body weight had an impact on the peak amplitude and dominant frequency of the VGRF spectrum, the data pertaining to each subject were plotted against their corresponding body weight. A linear regression model was used to study these relationships in the different visual and base of support conditions and in the non-adapted and adapted trials.

The HGRFs spectrum profile did not have the same bell shape as that of the VGRF spectrum, so the median frequency (at which the power spectrum is divided into two parts of equal area) and the area of the surface below the profiles of the HGRF spectra (i.e., the sum of the spectrum amplitude at each frequency sample) were calculated. A LabVIEW software routine was used. These variables were calculated for both the ML and the AP directions in the different vision and base of support conditions and for the non-adapted and adapted trials.

The frequency spectra of the CoP time series were compared to those of the HGRFs. For each subject and condition, the spectra (having different amplitude and unit of measurement) were normalised to their maximum value and then averaged across subjects. The mean power spectra profiles thus obtained were point-to-point compared by Student’s *t*-test according to a procedure already used [8]. The frequencies at which the *t*-test (two-tailed pairwise test) value was above the probability of 0.05 were taken as the frequencies at which CoP and HGRFs spectra had similar amplitudes. The frequency corresponding to the greatest probability was the intersection point of the two spectra.

### 2.4. Time–Domain Analysis

For each subject, condition and for both non-adapted and adapted trials, the path length of the CoP displacement in the horizontal plane was the total length calculated from the entire time series (90 s). In a similar way, the total variation of the HGRF was the sum of the differences between the forces recorded at two consecutive time-instants. Hence, the total variation of HGRF was:

$$\overset{90}{\underset{t=1}{\sum }}\sqrt[{2}]{\left|\left(MLGRF_{t}{}^{2}+APGRF_{t}{}^{2})-(MLGRF_{t-1}{}^{2}+APGRF_{t-1}{}^{2}\right)\right|}$$

The relationship between the total variation of the HGRF and of the path length of the CoP was assessed by plotting the data of all subjects, one against the other. These relationships were studied by a linear regression model and the R^2^ was calculated.

The similarity of the two HGRFs and CoP time series (for both AP and ML) was studied for each participant and for each condition by the cross-correlation (CC) analysis. The CC coefficient (*R*) at the time lag of zero s was calculated by means of the CC routine of the software Origin. A positive coefficient indicates in-phase change of forces and CoP positions, a negative coefficient indicates anti-phase change.

The HGRF amplitudes were also plotted as a function of the CoP positions during a trial as follows. For each subject and condition, CoP positions along both the ML and AP directions were normalised to their maximum excursion. This was done because the amplitude of the CoP excursions were different in the individual subjects. The absolute value of the amplitude of the HGRF was then averaged within consecutive bits, each corresponding to a 10% segment of the CoP maximum excursion (for both ML and AP directions). Then, the mean amplitudes of the HGRF thus obtained for each 10% segment of CoP excursion were averaged across the subjects.

### 2.5. Statistical Analysis

The minimum effect size was calculated using the G*Power 3.1.9.4 software [49]. Given our sample (n = 24), the study proved to have sufficient power (>80%) to detect an effect size (Cohen’s *d*) of 0.46. Assumptions for parametric statistics were met for all the variables of interest, as assessed by the Kolmogorov–Smirnov and Levene’s tests. The effects of adaptation, visual and base of support conditions on VGRF dominant frequency, VGRF peak amplitude, SD of the Gaussian fit, HGRF total force variation and CoP Path Length were compared by 2 (non-adapted or adapted trial) × 2 (vision conditions) × 2 (solid or foam) repeated measure (rm) ANOVA. A 2 (ML or AP directions) × 2 (non-adapted or adapted trial) × 2 (vision conditions) × 2 (solid or foam) rm ANOVA was used to compare the median frequencies. The HGRF and VGRF frequencies were compared by a 3 (force directions) × 2 (non-adapted or adapted trial) × 2 (vision conditions) × 2 (solid or foam) rm ANOVA. The CC coefficients, after z-transformation, were compared by a 2 (ML or AP directions) × 2 (non-adapted or adapted trial) × 2 (vision conditions) × 2 (solid or foam) rm ANOVA. The η^2^_p_ was reported as a measure of effect size. Post hoc analysis was performed using the Tukey’s HSD test. The significance level was set at *p* = 0.05. Statistical tests were performed using the software Statistica (TIBCO^®^ Data Science, D).

## 3. Results

Frequency and amplitude of the VGRF are presented first, followed by the results of the analysis of the HGRFs along the antero-posterior and medio-lateral axes and by the comparison of the changes in HGRFs and CoP oscillations. The effect of adaptation has been addressed for all variables and conditions.

### 3.1. The Vertical Component of the Ground Reaction Force (VGRF)

**Figure 1:**
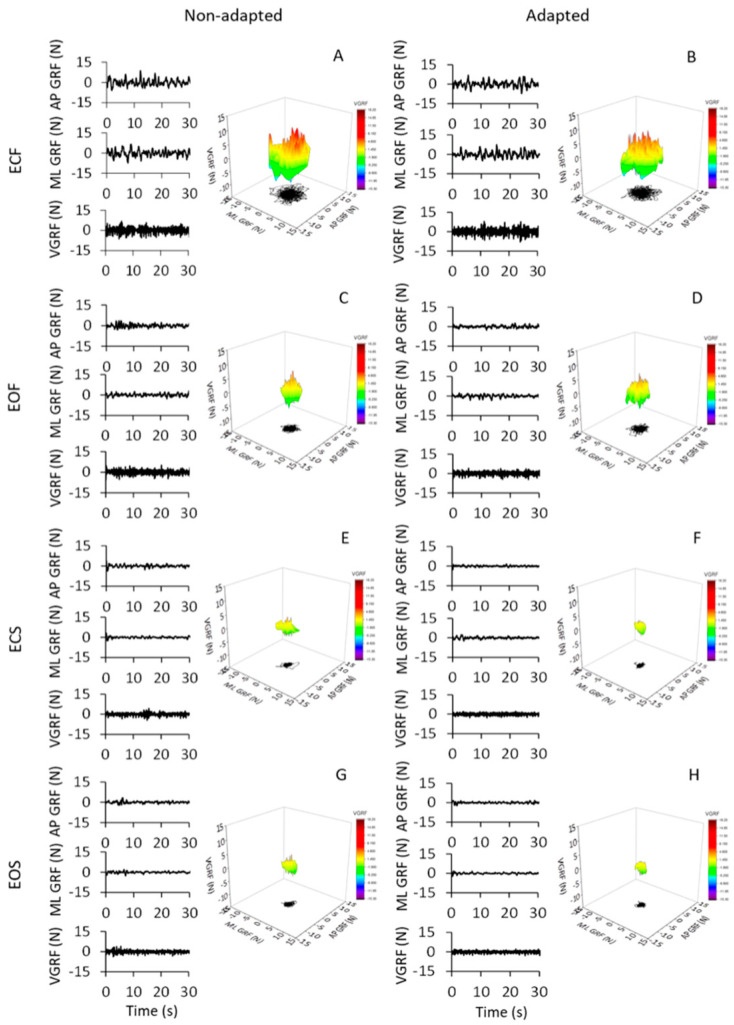
GRF oscillations in the vertical (VGRF) and horizontal (HGRF) planes. All panels (left columns), the time series of VGRF and the ML and AP HGRF oscillations are displayed during the first 30 s of acquisition for clarity. The data were recorded in a typical subject for the eyes closed (EC) and eyes open (EO) conditions on foam (F) and solid (S) base of support, during non-adapted trials (left side of the figure) and adapted trials (the last trials of a series of eight) (right side). The data of the entire duration of each trial were reported in the 3D graphs in (**A**–**H**). Zero in the ordinate for the VGRF corresponds to body weight. The GRFs (both VGRF and HGRF) were the largest in ECF (**A**,**B**), decreased with EOF (**C**,**D**) and were the smallest with the solid base of support (**E**–**H**). Adaptation had little effect in the ECF condition and hardly any effect in the others.

The oscillations of the vertical component of the VGRF of the subjects standing upright on the force platform varied in an ample range, depending on the availability of vision, the quality of the support surface and the process of adaptation to repeated stance trials. Figure 1 shows the VGRF oscillation traces and the corresponding ML and AP HGRF oscillation traces recorded in the first (non-adapted, left side of the Figure) and last trials (adapted, right side) of a typical subject, in the four different experimental conditions (ECF in Figure 1A,B, EOF in Figure 1C,D, ECS in Figure 1E,F and EOS in Figure 1G,H). Only the first 30 s have been shown here (the time series on the left part of each panel) out of the entire trial duration (90 s) for clarity of representation.

The data of the entire duration of each trial are represented in the nearby 3D graphs. The ECF condition was characterised by the greatest VGRF changes in amplitude around the value of the body weight (corresponding to the zero of the ordinates) and by the greatest HGRF omnidirectional displacement with respect to the other conditions. The order of magnitude of the force changes was the same for both the ML and the AP directions and there were no great differences between the two axes. The VGRF and HGRF oscillations decreased with EO and were the smallest with the solid base of support, in which case both forces had similar amplitudes between EC and EO conditions. With adaptation, the VGRF amplitude was reduced compared to the first trials, in particular with eyes closed on the foam support (ECF) (Figure 1B).

### 3.2. The Dominant Frequency of the VGRF Oscillation

**Figure 2:**
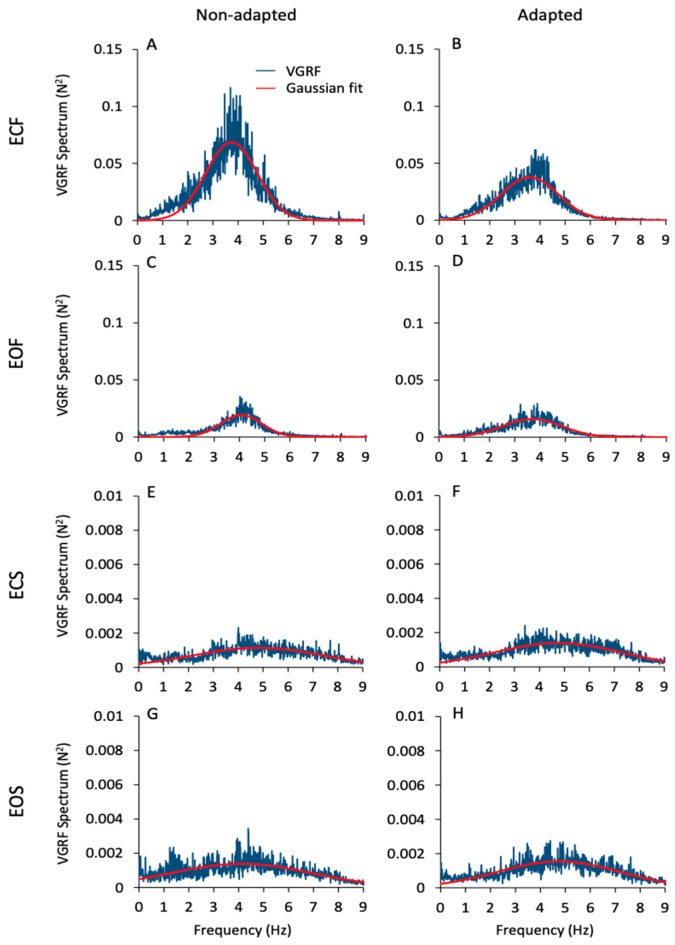
VGRF spectrum. The mean VGRF spectrum (blue traces) calculated across all subjects are reported for the ECF (**A**,**B**), EOF (**C**,**D**), ECS (**E**,**F**) and EOS (**G**,**H**) conditions for the non-adapted (left side of the figure) and the adapted trials (right side). The superimposed red traces are the Gaussian curves fitting the VGRF spectra. VGRF spectrum amplitude diminished with EO and with solid base of support. There was no difference between EC and EO conditions. The adaptation process diminished the spectrum amplitude only in the ECF condition. The dominant frequencies are similar between vision and support conditions and between non-adapted and adapted trials. Note the 15× ordinate values in (**A**–**D**) compared to (**E**–**H**).

Figure 2 gives an overall view of the mean frequency spectra of the VGRF oscillations. These are the average traces of the spectra of all subjects in all conditions (ECF, EOF, ECS, EOS, from top to bottom) prior to and after adaptation (left and right columns, respectively). Of note, the profiles of the spectra feature a probabilistic distribution of frequencies with a typical bell curve appearance. For this reason, a Gaussian curve (the red trace superimposed to the blue spectra) was fitted to the spectra (see Section 2). The mean value of the Pearson’s R coefficient of the fit was about 0.6 for the foam conditions (both EC and EO) and about 0.3 for the solid conditions (EC and EO) for both the non-adapted and the sdapted trials. There were large differences in the spectra profiles across conditions. The peak amplitude (ordinate, N^2^) was about 40-times larger for ECF than EOS (note the different ordinate values for the foam and solid panels). The dominant frequency (Hz) was taken as the value of the abscissa at the peak of the bell curve. The most represented frequencies ranged between approximately 2 Hz and 5 Hz when standing on the foam support, independent of vision condition. Frequencies higher than 6 Hz were negligible. However, frequencies from 6 Hz to 8 Hz were also observed on the solid-support condition, even if their amplitude was always below the amplitude corresponding to the dominant frequency. Despite the large divergence in peak amplitudes across conditions, the dominant frequencies were broadly similar under both vision and both support conditions and similar between non-adapted and adapted trials.

### 3.3. Modulation of Peak Amplitude and Dominant Frequency of the VGRF Spectrum by Vision, Support Surface, and Adaptation

**Figure 3:**
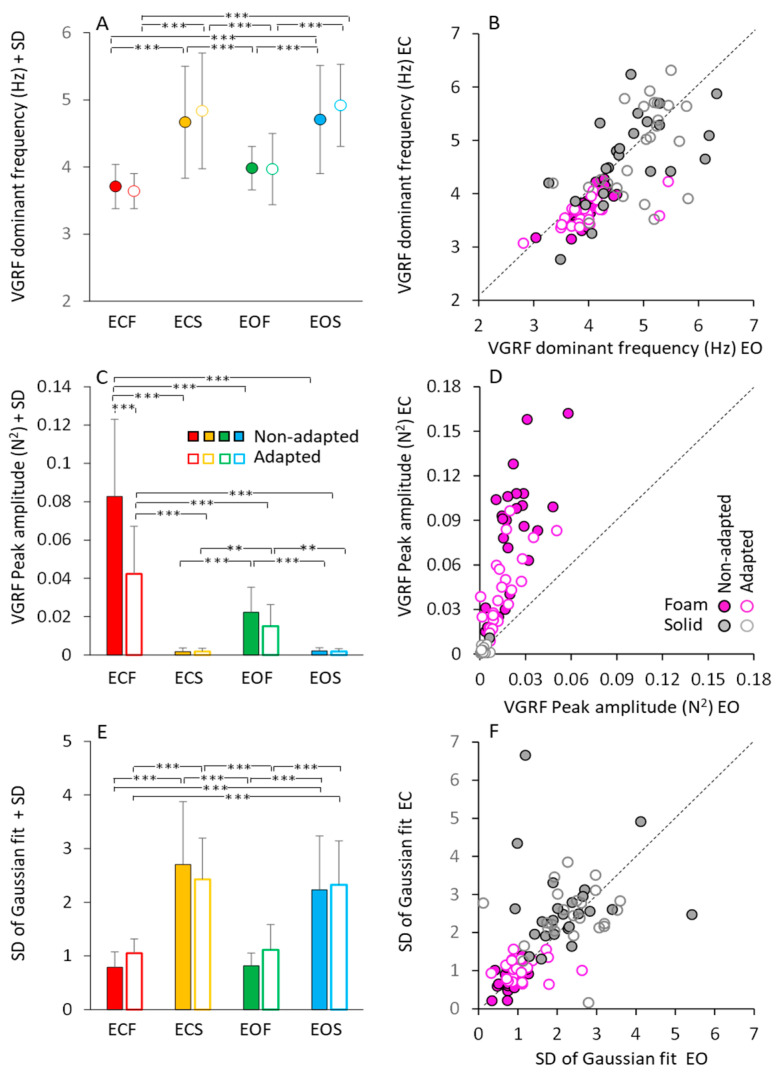
Gaussian fit parameters. The mean VGRF dominant frequency (**A**,**B**), mean peak amplitude (**C**,**D**) and mean SD of the Gaussian fit (**E**,**F**) calculated across subjects, are reported for the ECF (red), ECS (yellow), EOF (green) and EOS (blue) conditions in the non-adapted (filled bars and symbols) and adapted (empty bars and symbols) trials. In (**B**,**D**,**F**), the Gaussian fit parameters obtained under EC condition are plotted against the corresponding parameters obtained under EO, separately for the foam (pink filled and empty symbols) and solid condition (grey filled and empty symbols). Each symbol in (**B**,**D**,**F**) corresponded to a subject. The black dotted lines in (**B**,**D**,**F**) are the identity lines. VGRF dominant frequency and SD are higher on solid than on foam, but there is no difference between EC and EO and between non-adapted and adapted trials. Peak amplitude is the greatest in the ECF condition and decreased with EOF. It was the smallest with solid support, where vision had no effect. With adaptation, the peak amplitude decreased only with EC and foam base of support. Asterisks indicate significant differences ** *p* < 0.01; *** *p* < 0.001).

Figure 3 shows a summary of the mean profile characteristics of the VGRF frequency spectra (dominant frequency, peak amplitude, standard deviation) obtained by averaging the parameters of the bell curves fitted to the data of each subject. Although not much dissimilar across conditions, as seen in Figure 2, the mean dominant frequencies of the spectra showed moderate but significant differences.

Figure 3A shows that these frequencies were higher on solid (from 4.5 to 5 Hz, on average) than foam support (just below 4 Hz, on average) (main effect of base of support, F(1,23) = 86.24, *p* < 0.001, η^2^_p_ = 0.79). There was a modest difference in the mean dominant frequency between visual conditions (main effect, F(1,23) = 10.04, *p* < 0.01, η^2^_p_ = 0.30). However, the post hoc analysis found no difference between EC and EO for either foam (*p* = 0.45 for non-adapted, and *p* = 0.25 for adapted trials) or solid base of support (*p* = 0.99, for both non-adapted and adapted trials). Adaptation (open symbols) had no effect on the dominant frequency of the VGRF oscillations (main effect, F(1,23) = 1.47, *p* = 0.23, η^2^_p_ = 0.06), regardless of vision and support surface conditions.

To graphically demonstrate these relationships, all individual data points for the dominant frequencies are represented in Figure 3B, where the EC and EO data (plotted in ordinate and abscissa, respectively) are distributed along the identity line (representing equal dominant frequencies in both visual conditions) in spite of a moderate scatter of the data points. This was true for both foam and solid support (grey and pink circles).

Figure 3C shows the peak amplitude (in N^2^) of the mean dominant frequency of the VGRF spectra. Amplitude was much larger on foam (red and green) than on solid support (yellow and blue) (main effect, F(1,23) = 108.64, *p* < 0.001, η^2^_p_ = 0.83). There was a difference between vision conditions (main effect, F(1,23) = 99.82, *p* < 0.001, η^2^_p_ = 0.81), with an interaction between vision and base of support conditions (F(1,23) = 107.23, *p* < 0.001, η^2^_p_ = 0.82). Vision compared to no-vision clearly diminished the peak spectrum amplitude on foam (post hoc, *p* < 0.001 for both non-adapted and adapted trials), whereas the effect was absent on solid support (*p* = 1, for both non-adapted and adapted trials), where the spectra were a tiny fraction of those on foam. The effect of adaptation on the amplitude of the spectra was significant as well (main effect, F(1,23) = 37.58, *p* < 0.001, η^2^_p_ = 0.62). There was an interaction between adaptation and vision (F(1,23) = 22.43, *p* < 0.001, η^2^_p_ = 0.49) and between adaptation and base of support conditions (F(1,23) = 37.2, *p* < 0.001, η^2^_p_ = 0.62). On foam, adaptation reduced the VGRF peak amplitude with EC (post hoc, *p* < 0.001) but not with EO (*p* = 0.94). Adaptation was instead ineffective on the solid support under both visual conditions (*p* = 1, for both comparisons). In Figure 3D, the steep slope for the foam condition (ECF plotted versus EOF for all subjects) pointed again to a large effect of vision on foam support for both non-adapted and adapted condition.

Figure 3E,F show that the bell curves had a significantly larger dispersion (SD) on dolid (yellow and blue bars in Figure 3E and grey circles in Figure 3F) than on foam support (red and green bars in Figure 3E and pink circles in Figure 3F) (main effect, F(1,23) = 188.96, *p* < 0.001, η^2^_p_ = 0.89). This indicates a non-negligible contribution to the VGRF spectra in the solid support condition by oscillations frequencies lower or higher than the dominant frequency. Adaptation (open bars in Figure 3E) (main effect, F(1,23) = 1.09, *p* = 0.3, η^2^_p_ = 0.04) and vision (Figure 3F) (main effect, F(1,23) = 1.43, *p* = 0.24, η^2^_p_ = 0.06) were ineffective on the SD value of the bell curves. Table 1 reports the equations of the regression lines (second column) fitting the EC vs. EO data points for dominant frequency (data reported in Figure 3B), peak amplitude (Figure 3D) and SD of the Gaussian fit (Figure 3F), the R^2^ (third column), the *p*-value of the statistical comparison between the slope of the regression lines and zero (fourth column). It also reports the statistical comparisons of the equations of the regression lines for the non-adapted and adapted trials (last column of the Table 1). Thus, vision, support surface and adaptation modified the amplitude of the VGRF spectrum. The solid support (moderately) increased the dominant frequency of the VGRF spectrum and broadened the spectrum while decreasing its overall amplitude. Under all conditions, adaptation did not materially affect the dominant frequency, or the range of the frequencies, represented in the spectrum.

**Figure 4:**
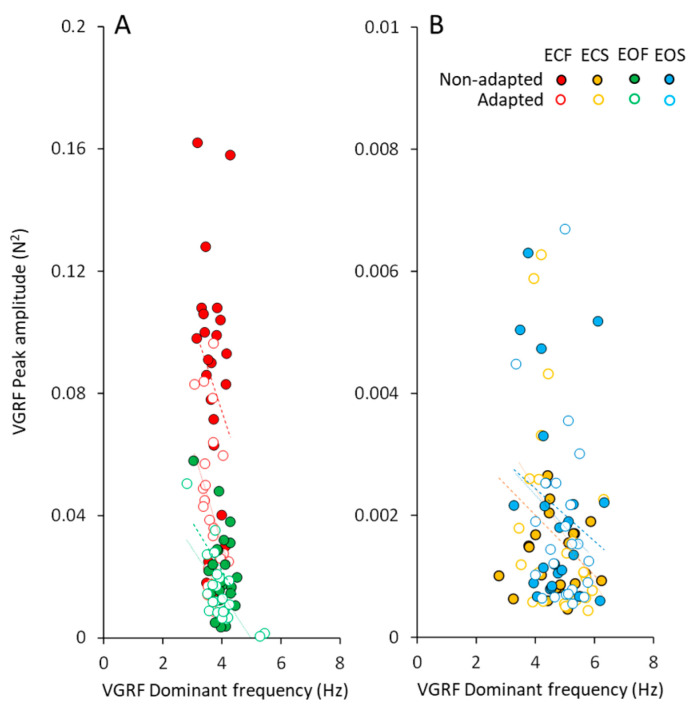
VGRF peak amplitude vs. dominant frequency. The VGRF peak amplitude was plotted against the corresponding value of VGRF dominant frequency under foam (**A**) and solid (**B**) support conditions (ECF, red; ECS, yellow; EOF, green and EOS, blue) in the non-adapted (filled symbols) and adapted trials (open symbols). Each symbol corresponded to a subject. With foam support (**A**) the dominant frequency varied in a narrow range despite a large change in the peak amplitude of the spectrum across subjects. With solid support (**B**), the dominant frequencies were scattered in a larger range with respect to foam condition, but their peak amplitudes were much smaller (note the different scales on the y-axis). For both foam and solid, the data points of the adapted trials (open symbols) are dispersed among the filled ones.

The peak amplitudes of the VGRF spectra plotted against the corresponding dominant frequencies are shown in Figure 4, for all subjects. In spite of a remarkable scatter of the data points (ordinate, from about zero N^2^ to more than 0.16 N^2^), the changes in the corresponding dominant frequency were restricted in a narrow range. This was obvious for both vision conditions on foam support. The data of the adapted trials (open circles) were mixed-up with those of the non-adapted condition, showing again that adaptation did affect the amplitude but not the dominant frequency of the VGRF. The very small spectrum amplitudes observed with solid support were scattered across a wider range of the dominant frequencies.

### 3.4. Body Weight Modifies Both Frequency and Amplitude of the VGRF Spectrum

**Figure 5:**
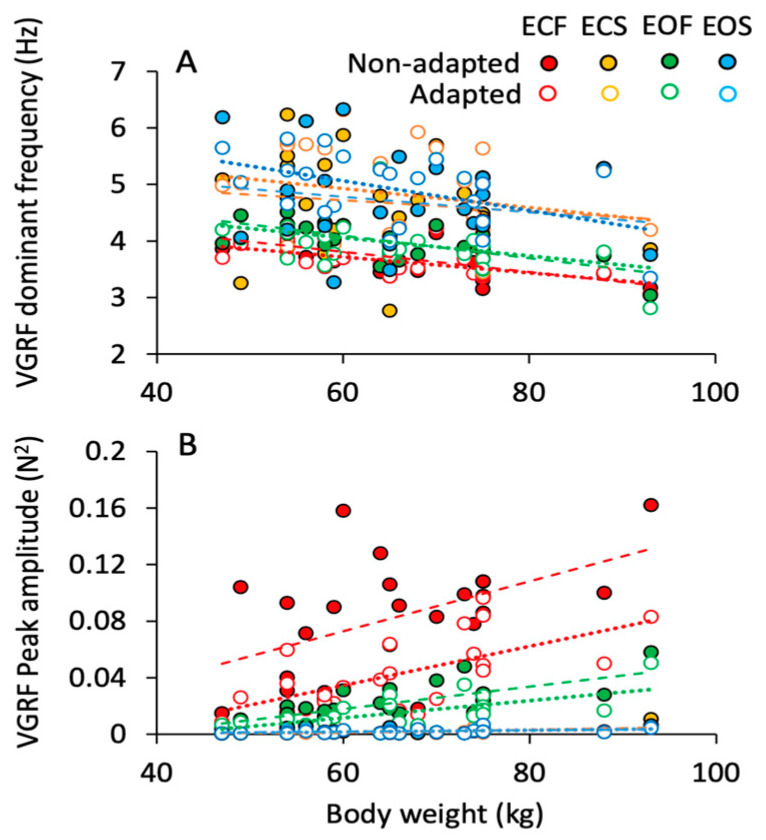
Effect of body weight on the VGRF dominant frequency and peak amplitude. The VGRF dominant frequency (**A**) and peak amplitude (**B**) of each subject are plotted against the corresponding body weight for the ECF (red), EOF (green), ECS (yellow) and EOS (blue) conditions, for both the non-adapted (filled symbols) and adapted (empty symbols) trials. Each symbol corresponds to a subject. Dashed lines are fitted on the non-adapted data, dotted lines to the data of adapted trials. VGRF dominant frequency showed a slight decrease with increasing body weight under all conditions. The peak amplitude, instead, increased with increasing body weight with foam base of support but not with solid, where body weight had no effect.

The effects of body weight on the VGRF frequency and amplitude are shown in Figure 5A,B. The dominant frequency of the spectrum (Figure 5A) decreased by less than 1 Hz across the subjects with smaller to bulkier body frame. This appeared to be common to all conditions. On the other hand, the peak amplitude of the spectrum (Figure 5B) more than doubled with body weight. This effect was obvious when standing on foam regardless of vision (green, EO; red, EC) and adaptation (open symbols). On solid support, the relationship between spectrum amplitude and weight was not influenced by vision or adaptation. The equations of the regression lines fitting the relationship between VGRF dominant frequency and body weight and between VGRF peak amplitude and body weight are reported in Table 2 and Table 3, respectively. 

The *p*-value of the comparison between the slope of the regression lines and zero are reported in the fourth column of the tables. In the last two columns, the slopes of the regression lines are compared (the *p*-values are reported) between non-adapted and adapted trials and between conditions separately for the non-adapted and adapted trials.

### 3.5. Frequency and Amplitude of the Horizontal Ground Reaction Force (HGRF) Do Not Match Those of the VGRF

**Figure 6:**
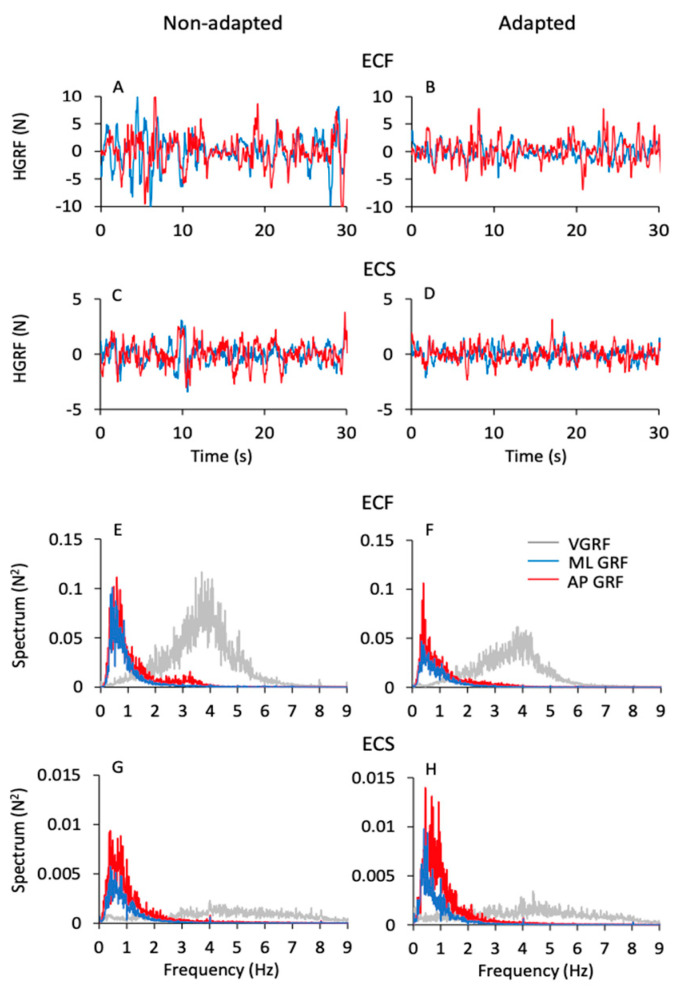
HGRF time series and frequency spectra. (**A**–**D**) show the HGRF oscillations along the ML (blue traces) and AP (red traces) directions of a typical subject under the ECF (**A**,**B**) and ECS (**C**,**D**) conditions for the non-adapted (left side) and adapted (right side) trials. HGRF oscillation amplitudes diminished with solid base of support (note the different scale between (**A**,**B**) and (**C**,**D**) panels) and with adaptation. (**E**–**H**) show the mean spectra calculated across subjects of the ML HGRF (blue traces) and AP HGRF (red traces) superimposed to the mean spectra of the VGRF (light grey traces) in the ECF (**E**,**F**) and ECS (**G**,**H**) conditions for both non-adapted (left side) and adapted (right side) trials. The median frequency of the HGRFs (both ML and AP) is around 0.5 Hz, while the VGRF dominant frequency is around 4 Hz.

Figure 6 shows the time series of the HGRF oscillations (Figure 6A–D) along the antero-posterior (AP, red traces) and the medio-lateral (ML, blue traces) directions in one subject. The panels refer to the ECF and ECS conditions for both non-adapted and adapted trials. As in Figure 1, only 30 s of the acquisition epoch are reported for clarity. The HGRF oscillations were larger with foam (Figure 6A,B) than solid support (Figure 6C,D) (note the different scale between Figure 6A,B and Figure 6C,D) and diminished with adaptation. In the lower part of the Figure (Figure 6E–H), the average frequency spectra of the HGRF oscillations along the antero-posterior (AP, red traces) and the medio-lateral directions (ML, blue traces) are shown. They are superimposed to the average spectrum of the VGRF (light grey traces) of the corresponding conditions in the non-adapted (Figure 6E,G) and adapted trials (Figure 6F,H). Again, note the different scale of the ordinates.

While the VGRF spectrum had a peak around its dominant frequency of 4 to 5 Hz, the oscillations of the HGRF (both ML and AP) had much lower frequencies overall, with a peak around 0.5 Hz. Both HGRF frequency spectra featured ample low-frequency values. Spectrum amplitude diminished gradually as a function of the increase in frequency, both with and without adaptation. The oscillations almost vanished beyond 4–5 Hz, so that the asymmetric profiles of the spectra were not fitted by a bell curve. Hence, the most represented frequencies of the VGRF were not represented in the spectrum of the HGRFs, which is basically shaped by low-frequency oscillations.

**Figure 7:**
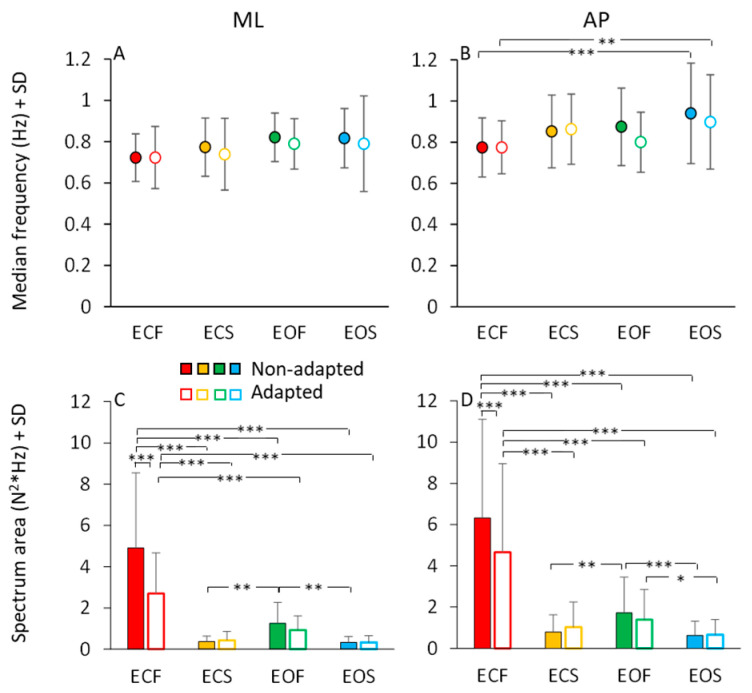
HGRF median frequency and spectrum area. The mean median frequency across subjects of the ML HGRF (**A**) and AP HGRF (**B**) are slightly higher with solid base of support and with EO. Adaptation has no effect on the median frequency of HGRFs. Spectrum area (ML, (**C**) and AP, (**D**)) decreases with vision and solid base of support. Adaptation reduces the HGRF spectrum area only in the ECF condition for both ML and AP directions. Red (dots and bars) refer to ECF, yellow to ECS, green to EOF and blue to EOS. Filled dots and bars refer to non-adapted trials and empty dots and bars to the adapted trials. Asterisks indicate significant differences (* *p* < 0.05; ** *p* < 0.01; *** *p* < 0.001).

In Figure 7, the mean values of the median frequency (Figure 7A,B) and of the area of the HGRF (AP and ML) spectra (Figure 7C,D) are reported for each visual and base of support condition in the non-adapted (filled dots and bars) and adapted trials (open dots and bars). The mean values of the median frequency of the HGRF oscillations were below 1.0 Hz under all conditions. As expected by observation of Figure 6, the VGRF and HGRF frequencies were significantly different (main effect, F(2,46) = 1450.77, *p* < 0.001, η^2^_p_ = 0.98) (in this statistical comparison, we arbitrarily compared the dominant frequency of VGRF with the median frequency of the HGRFs).

When we specifically considered the median frequencies of the AP and ML components of the HGRF, we found a significant main effect of ML and AP directions, the median frequency being about 10% higher in AP than ML (main effect, F(1,23) = 10.09, *p* < 0.01, η^2^_p_ = 0.3). There were modest but significant differences in the median frequencies between visual conditions (EO > EC; main effect, F(1,23) = 13.37, *p* < 0.01, η^2^_p_ = 0.37) and between support conditions (solid > foam, main effect, F(1,23) = 5.72, *p* < 0.05, η^2^_p_ = 0.19). There was an interaction between ML or AP direction and support condition (F(1,23) = 4.96, *p* < 0.05, η^2^_p_ = 0.17), because the AP median frequency was greater with solid than with foam base of support. Adaptation was ineffective on the median frequency of the HGRF in both AP or ML directions within each condition (main effect, F(1,23) = 2.07, *p* = 0.16, η^2^_p_ = 0.08). Hence, modest changes in the median frequency of HGRF oscillation were observed, with a slight tendency towards higher frequencies in the more ‘safe’ condition (EOS). The median frequency was greater only in the AP direction in EOS than ECF condition for both non-adapted and adapted trials (*p* < 0.01 for both comparisons). Overall, the quality of these frequency variations as a function of experimental conditions are consistent with those observed for the VGRF (compare Figure 7A,B with Figure 3A).

The area of the surface below the profiles of the HGRF spectra (Figure 7C,D) was larger with EC than EO (main effect, F(1,23) = 43.02, *p* < 0.001, η^2^_p_ = 0.65), with foam than solid support (main effect, F(1,23) = 43.16, *p* < 0.001, η^2^_p_ = 0.65) and in AP than ML direction (main effect, F(1,23) = 11.20, *p* < 0.01, η^2^_p_ = 0.33). There was a significant interaction between direction and visual conditions (F(1,23) = 7.10, *p* < 0.05, η^2^_p_ = 0.24) because vision reduced the area more in the AP than in the ML direction.

Adaptation did not affect the median frequency of the HGRF spectrum, but reduced the amplitude of the oscillations (F(1,23) = 17.05, *p* < 0.001, η^2^_p_ = 0.43). There was a significant interaction between adaptation and vision (F(1,23) = 10.65, *p* < 0.001, η^2^_p_ = 0.32) and between adaptation and base of support conditions (F(1,23) = 26.80, *p* < 0.001, η^2^_p_ = 0.54). Adaptation had a significant effect only in the ECF condition for both ML and AP directions (post hoc, *p* < 0.001 for both comparisons). With EO on foam and with both EC and EO on solid base of support, there was no difference between the area under the spectrum of the non-adapted and adapted trials (*p* > 0.85 for all comparisons). Overall, the effects of the conditions were similar on the spectral amplitudes of both HGRF and VGRF (compare Figure 7C,D with Figure 3C).

### 3.6. The Frequency Spectra of the Horizontal Components of the HGRF (ML and AP) Do Not Reflect Those of the CoP Oscillations

**Figure 8:**
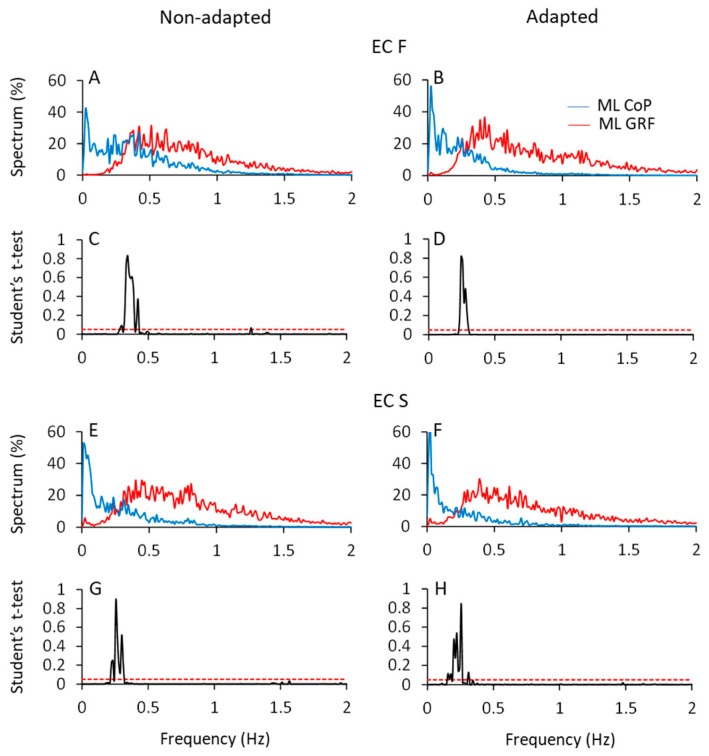
CoP and HGRF spectra in the ML direction. In (**A**,**B**,**E**,**F**), the normalised mean spectra of the ML CoP displacement (blue traces) and HGRF oscillations (red traces) are superimposed for the non-adapted (left side of the figure) and adapted (right side) trials of the ECF (**A**,**B**) and ECS (**E**,**F**) conditions. (**C**,**D**,**G**,**H**) show the point-to-point comparisons of the two spectra by means of the Student’s t-test. The two spectra crossed over at about 0.3 Hz.

Figure 8 shows the mean profiles of the spectra of the HGRF and CoP oscillations in the ML direction (AP not shown), with eyes closed on foam (ECF, top part of the Figure) and solid support (ECS, bottom part) before (left column) and after adaptation (right column). All the spectra were computed on the normalised traces to ease comparison (see Section 2).

A fair portion of the frequency content of HGRF spectra lay in the range between 0.3 Hz and 1.5 Hz, while the frequency content of the CoP was comprised between approximately 0.01 Hz and 0.6 Hz, for both ML and AP directions. The CoP spectra featured ample values at low frequency and decreased amplitude with increasing frequency. The HGRF spectra showed almost no very low-frequency content but relatively ample values for frequencies from 0.2 Hz to 1.5 Hz, approximately. For example, in the ECF condition, the point of intersection of the 2 traces separated the CoP and HGRF spectra into 2 parts of low and high frequencies, the former containing only 8% of the HGRF spectrum and 50% of the CoP spectrum. Hence, the most represented frequencies of the HGRF oscillations did not transfer onto those of the CoP.

The HGRF and CoP traces were compared point-to-point by the Student’s *t*-test. Figure 8C,D,G,H indicate the crossing points of the average profile of the spectra. The probability spikes (*p* > 0.05) of the point-to-point *t*-test comparison between the 2 mean traces indicate the frequencies at which the intersection occurred. Overall, adaptation minimally diminished the frequency at the intersections. The median frequency of HGRF did not seem to change with adaptation and conditions, contrary to that of CoP [10]. The intersection frequencies (see Table 4) were just higher with foam than solid support and moved toward low frequency values passing from the more unstable (ECF) to the more stable conditions (ECS and EOS). Thus, the shift in the intersection points depends on changes in the CoP spectrum rather than in those of the HGRF.

### 3.7. The Cross-Correlation between the Oscillations of the HGRF and Those of the CoP

**Figure 9:**
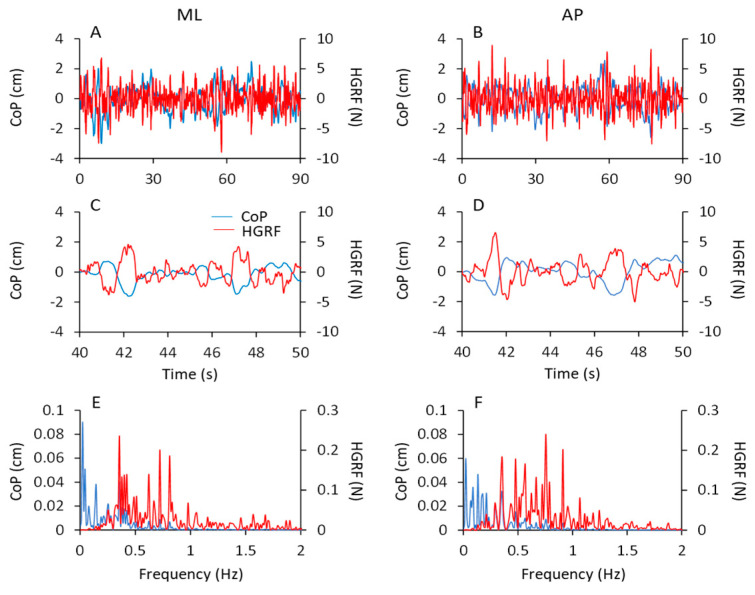
CoP and HGRF oscillations. In (**A**,**B**) the CoP displacement (blue traces) and HGRF oscillations (red traces) of a typical subject are superimposed for the ML (left side of the figure) and AP (right side) direction during the first trial in ECF condition. In panels (**C**,**D**), only 10 s have been shown, for clarity of representation. CoP and HGRF signals showed an anti-phase displacement. The corresponding spectra of the CoP and HGRF signals are reported in panels (**E**) (ML direction) and (**F**) (AP direction). Between 0.3 and 0.5 Hz both the CoP and HGRF spectra showed a considerable amplitude.

Figure 9 shows the data of a typical subject in the ECF condition. The time series of the ML and AP HGRF values (red traces in Figure 9A,B) and of the CoP positions (blue traces) are plotted for the entire acquisition epoch. In Figure 9C,D, 10 s of the traces are unfolded to compare the traces easily. The excursions of CoP and HGRF are necessarily expressed in different units (blue, cm and red, N, respectively), so that any comparison of the amplitudes of the graphed displacements is unwarranted. CoP and HGRF clearly showed opposite-phase variations with respect to each other (Figure 9C,D) both in the ML and the AP directions, where a positive peak in the CoP traces corresponded to a negative peak in the HGRF plot. When CoP moved to the right or forwards (positive values on the ordinates), the corresponding HGRFs were directed to the left or backwards (negative values). Vice versa when CoP is placed to the left or backward with respect to its mean position (negative values on the ordinates) the corresponding HGRFs were directed to the right or forward (positive values).

In the bottom panels, the corresponding spectra of the CoP (blue traces) and HGRF (red traces) signals are reported for both ML (Figure 9E) and AP (Figure 9F) directions. The peaks of the CoP spectrum (both ML and AP) had a large amplitude in the frequency range of 0.01–0.5 Hz and decreased at higher frequencies. The HGRF spectrum showed instead large amplitudes between 0.3 and 1 Hz, and small amplitudes for frequencies below 0.3 Hz and above 1 Hz. A common window of frequencies (approximately 0.2 Hz to 0.4 Hz) exhibited considerable amplitude in both CoP and HGRF spectra.

The HGRF and CoP time-series have been compared by a simple cross-correlation (CC) analysis, for both the ML and the AP directions. In spite of the remarkably different frequency content of the mean spectra shown above, the oscillations of HGRF and CoP showed fair CC coefficients. Table 5 reports the mean values of the coefficients averaged from all the subjects in the four conditions. The mean values ranged from −0.45 ± 0.15 to −0.76 ± 0.08 for the ML direction and from −0.43 ± 0.12 to −0.74 ± 0.06 for the AP direction. For all conditions and subjects, the coefficients were negative indicating that CoP and HGRF oscillations were in opposition of phase. The relatively high coefficients are therefore justified by the frequency window common to the two spectra.

Overall, the mean coefficients were significantly larger for non-adapted than adapted trials (main effect, F(1,23) = 47.73, *p* < 0.001, η^2^_p_ = 0.67), for EC than EO condition (main effect, F(1,23) = 207.33, *p* < 0.001, η^2^_p_ = 0.67), for foam than solid support (main effect, F(1,23) = 66.33, *p* < 0.001, η^2^_p_ = 0.74). There was a modest but significant difference between ML and AP direction as well (main effect, ML > AP, F(1,23) = 12.01, *p* < 0.01, η^2^_p_ = 0.34).

### 3.8. The HGRFs Have an Ill-Defined Relationship with the Excursions of the CoP

**Figure 10:**
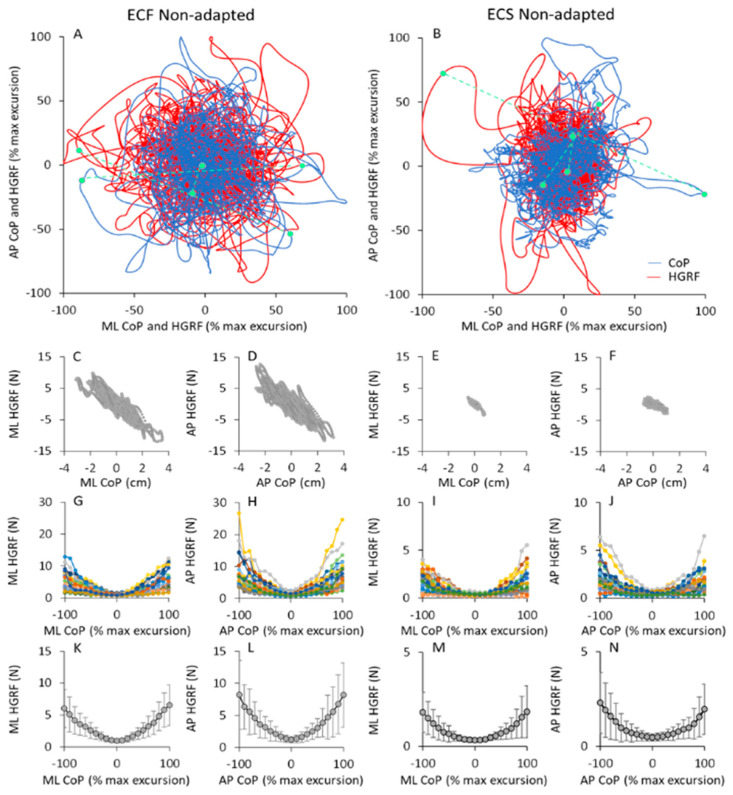
CoP and HGRF oscillations in the horizontal plane. In (**A**,**B**), the CoP displacement (blue traces) and HGRF oscillations (red traces) of a typical subject in the ML direction are plotted against the corresponding CoP and HGRF values along the AP direction during the first trial of the ECF (**A**) and ECS (**B**) conditions. All values are reported in % of the maximal excursion of the variables. The three small green symbols connected by dashed lines in (**A**,**B**) correspond to three CoP and HGRF samples recorded at the same time instant. In panels C to F, the HGRF values of the same subject are plotted against the corresponding CoP position for both ML (ECF, (**C**) and ECS, (**E**)) and AP directions (ECF, (**D**) and ECS, (**F**)). In panels G to H, the mean amplitude of HGRF calculated for each 10% change in the displacement of the CoP from its mean position are reported for all subjects (each coloured line corresponds to a subject) for both ML (**G**,**I**) and AP (**I**,**J**) directions. The mean and SD values across subjects are shown in panels K to N. The amplitudes of HGRF (in ML and AP directions) are greater the further the CoP moves away from its mean position (zero on the abscissa). This occurs with both foam and solid base of support. Note that the ordinates of (**I**,**J**,**M**,**N**) (solid condition) are three times smaller than those of (**G**,**H**,**K**,**L**) (foam condition).

In Figure 10A,B the trajectories of the HGRF and CoP of a typical subject in the ECF (Figure 10A) and ECS (Figure 10B) conditions (both non-adapted) are represented. The values corresponding to the ML and AP axes were plotted against each other for both HGRF and CoP data before being superimposed in the graphs.

Clearly, in both cases, there was a good match between the HGRF and CoP traces. Because of the different measurement units (red, N; blue, cm), the values of the HGRF and CoP traces were normalised here to their own maximum values (see Section 2) during the trial for the sake of easy comparison. For this reason, the plots appear to have similar dimensions, despite the real HGRF and CoP excursions being much smaller in ECS (right panel) than ECF conditions (left panel).

There was a tendency for both the HGRF and the CoP data points to be concentrated close to the origin of the plot, and to move with more or less ample and slow displacements around the boundaries of the area covered by the CoP oscillations. Three data points per condition (green symbols in Figure 10A,B) sampled at the same time instant for CoP and HGRF profiles are connected by green dashed lines to show examples of the concurrence. The symbols on the blue trace (CoP) correspond to the point of application of the HGRF value indicated by the symbol on the red trace. As already shown by the cross-correlation analysis between the two signals, CoP and HGRF were in phase opposition. For example, a CoP sample in the first quadrant (top, right) of the horizontal plane (i.e., a CoP position on the right and forward with respect to its mean position) corresponded to a HGRF sample in the third quadrant (i.e., to an AP HGRF directed backward and to a ML HGRF directed to the left). In addition, ampler CoP excursions (the points farther from the origin of the axes in the blue traces) corresponded to higher HGRF forces (the points farther from the origin of the axes in the red traces). This behaviour was common to both foam and solid support. In the latter case, the excursions of the HGRFs had a somewhat greater extent (in proportion of the entire width) than with foam.

In the second-row panels of the Figure 10, the HGRF data of the same subject in Figure 10A,B are plotted against the corresponding CoP positions, for both ML (Figure 10C,E) and AP directions (Figure 10D,F) in the ECF and ECS non-adapted trial. HGRF amplitudes and CoP excursions were greater with foam than solid base of support. The maximum CoP excursion was different between the two conditions. However, the graphs show that regardless of the conditions, the force amplitudes increased as the position of the CoP moved away from its mean position (zero on the abscissa), both in AP and ML directions, and that CoP and HGRF were regularly in opposition of phase.

The relationship between HGRF amplitude and CoP position is shown in the Figure 10G–J, where the mean amplitude of HGRF within each 10% segment of CoP displacement is plotted against the mean CoP position as a percentage of its maximum excursion. For all subjects, the HGRF amplitude was larger at the extreme positions of the CoP, both in AP and ML direction. This behaviour was common to all subjects and support conditions, despite large differences between subjects (shown by the family of coloured curves), particularly at the extremes of the CoP excursions. In the last row of the Figure 10K,N, the mean values across subjects are reported to highlight the bowl-shaped function of HGRF versus CoP.

### 3.9. Vision, Support Surface and Adaptation Equally Affect HGRF and CoP Excursions

**Figure 11:**
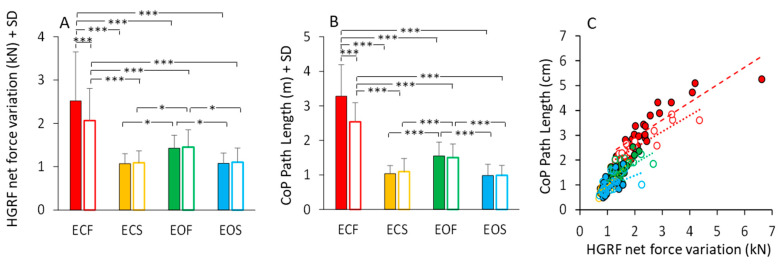
HGRF total force changes and CoP path length. HGRF total variation (**A**) decreased with vision (on foam, ECF > EOF) and solid base of support (no difference between ECS and EOS). Adaptation has an effect only in the ECF condition. The effects of the conditions on the HGRF total variation strongly match those on the CoP path length (**B**). (**C**) shows the relationship between the HGRF total variation and CoP path length in all the subjects (each symbol corresponds to a subject). Path length increased as a function of the increase in HGRF total variation, even if the ratio differed somehow across subjects. Red (bars end symbols) refer to ECF, yellow to ECS, green to EOF and blue to EOS. Filled bars and symbols refer to non-adapted trials and empty bars and symbols to the adapted trials. Asterisks indicate significant differences (* *p* < 0.05; *** *p* < 0.001).

The total HGRF variation over time was calculated for each subject and trial (see Section 2). The mean value is reported in Figure 11A. Anova showed a significant difference between non-adapted and adapted trial (main effect, F(1,23) = 11.36, *p* < 0.01, η^2^_p_ = 0.33) and between visual conditions (main effect, F(1,23) = 33.32, *p* < 0.001, η^2^_p_ = 0.59). With foam, the HGRF total force variation was greater than with solid base of support (main effect, F(1,23) = 63.96, *p* < 0.001, η^2^_p_ = 0.73). The mean values of the CoP Path Length in the different visual and support conditions in the first trial (filled bars) and after adaptation (empty bars) are reported in Figure 11B. Path length was long on foam and short on solid support (main effect of support, F(1,23) = 231.14, *p* < 0.001, η^2^_p_ = 0.91). There was a significant effect of visual condition (F(1,23) = 98.69, *p* < 0.001, η^2^_p_ = 0.44) and a significant interaction between visual condition and base of support (F(1,23) = 155.82, *p* < 0.001, η^2^_p_ = 0.87). Path Length was greater with EC than with EO only on foam (*p* < 0.001 for both non-adapted and adapted trials). The difference in path length between non-adapted and adapted trials was significant (main effect, F(1,23) = 18.12, *p* < 0.001, η^2^_p_ = 0.44). The adaptation effect (open bars in Figure 11B) was obvious only for the EC foam condition (post-hoc, *p* < 0.001).

Figure 11C shows that the length of the CoP oscillations closely matched the HGRF total net force variation. All conditions are depicted in this scatterplot. Despite the very different spectra between HGRF and CoP, there was an overall correspondence between CoP path length and total net force variation of HGRF, which approached a linear relationship across all conditions. The slopes of the straight lines (Figure 11C) plotted on the path length vs. force variation data were similar across the different conditions, supporting the conclusion that the length of the CoP displacement strictly, even if not entirely, depends on the total variation of the HGRF. Further, the slope of the relationship (Table 6) does not vary with the visual and support base conditions and with adaptation, indicating no separate effects of the conditions on the transfer function of the HGRF changes onto the CoP changes. The equations of the regression lines fitting the data points of the CoP path length vs. HGRF total net force variation (Figure 11C) are reported in Table 6. The equations of the regression lines are reported in the second column and the R^2^ in the third column. The *p*-value of the statistical comparison between the slope of the regression lines and zero are reported in the fourth column of the table. In the last two columns, the slopes of the regression lines are compared (the *p*-values are reported) between non-adapted and adapted trials and between conditions separately for the non-adapted and adapted trials.

## 4. Discussion

### 4.1. Recap and Outline of the Main Findings

The mechanisms underlying upright stance constitute a complex set of controls of the inherently unstable body structure. By and large, the CoP excursions on the base of support are ultimately produced by the combined action of the muscles and by the position of the Centre of mass (CoM) of the body, both of which directly modify the value of the ground reaction force (GRF). The continuously fluctuating GRF is the net result of the higher brain networks controlling posture of the feedback from diverse sensory pathways, including cutaneous afferent receptors, proprioceptors of numerous muscles, vision and vestibular sense [24,50] and of the muscle responses to instability acting onto the body’s mechanical constraints. We show here that a robust “postural rhythm” generator mayconstitute a neural constraint lying at the interface between internal and external events.

Here, we have addressed the frequency and amplitude of the oscillations of the vertical GRF (VGRF) [26,33,51] and the effects thereupon of the experimental conditions (vision, support surface, adaptation); then, we moved to the analysis of the horizontal GRF (HGRF) and compared the oscillation frequency of the latter to those of the CoP. Our premise was that a comparative analysis of the VGRF, HGRF and CoP features would help better clarify the origin of the CoP excursions, normally taken as a proxy of body stability, and better decipher how sensory conditions and adaptation ultimately impact on body sway. We have confirmed that the profile of the frequency spectrum of the VGRF has a dominant frequency between 3 Hz and 5 Hz (the “postural rhythm”) with a probabilistic frequency distribution [32], so that it is easily fitted by a Gaussian curve [10,33].

The frequency spectrum of the HGRF oscillation is quite different from that of the VGRF oscillations, and contains a much larger proportion of low frequency oscillations, with a skewed profile that declines gradually in amplitude at higher frequencies. The HGRF and CoP oscillation time-series exhibit fair anti-phase cross-correlation values, pointing to a distinct mechanical relationship between the two measurements. The CoP oscillations have a frequency content and distribution broadly evocative of those of HGRF, but still contain lower frequencies. The magnitude of the ML and AP HGRF variation increases with the distance of the CoP instantaneous position from its mean position, so that the HGRF values plotted as a function of the CoP position configure a bowl-shaped contour where the CoP appears to be a ball in the bowl.

Vision, support surface and adaptation affect the frequency distribution of the VGRF and HGRF spectra to a relatively small extent, revealing that the oscillations of VGRF and HGRF are poorly affected by the experimental conditions. This differs in the case of the CoP. In spite of a large overlap range of the HGRF and CoP spectra, the CoP median frequency is affected by experimental conditions, being the lowest in the more stable condition (EOS) and in the adapted trials [10,20].

### 4.2. The VGRF Frequency Undergoes Minor Changes between Compliant and Hard Support

Although not always explicitly, it has often been considered that a compliant support was simply a way to make standing critical or to mimic a sensory deficit, especially by making proprioceptive feedback less pertinent, mostly at the ankle [52,53,54]. However, increasing postural demand leads to an increase in activity of many muscles, including the intrinsic foot muscles [55]. On the other hand, recent data show that proprioception is not significantly associated with body sway while standing on foam with eyes closed [56,57]. That said, the use of hard and compliant support, as seen from a frequency perspective, can give insight into the complex organisation of the postural behaviour.

The *amplitude* of the oscillations of the VGRF around the body weight ranges from a maximum peak-to-peak value around ±20 N in the most critical condition (eyes closed on foam, ECF) to less than ±2 N (eyes open on solid, EOS), and broadly matches the values of the CoP geometrical features [33]. Oscillations are very small on the hard surface regardless of vision availability, where weak activity of leg and foot muscles is amply sufficient for body balancing around the vertical [58,59,60], and increase on the compliant surface, where removal of vision further increases oscillation amplitudes. The larger instability on foam produces greater hip and knee motion compared to ankle motion [61,62].

Across all experimental conditions, the dominant VGFR *frequency* of the VGRF bell-shaped spectrum moves within a much smaller range compared to the ample changes in spectrum amplitude. The dominant frequency lies between 3 Hz and 6 Hz, with lower and higher frequencies being almost absent below 2 Hz and above 8 Hz. More than 99% of the area of the spectrum profile is contained below 9 Hz, with both EC and EO and with both foam and solid support. Within these limits, the spectrum profile is much lower and broader on hard than compliant support, but the *dominant* frequency changes moderately. The dominant frequencies of the spectrum (larger by about 1 Hz on the hard compared to the compliant support, a difference observed in most individuals) are in line with the report by [38], who showed that the VGRF frequencies corresponding to the maximum power were 4.5 Hz and 3.2 Hz on hard and compliant support, respectively. The foam cushion would act mechanically as a low-pass filter by introducing a time constant between the development of the muscle torques and the recorded force, thereby modifying the fundamental frequency of the postural rhythm [38].

On hard compared to compliant support, the dominant frequencies are more variable across subjects and each subject oscillates within a broader range of frequencies. A rhythm between about 4 and 8 Hz while standing on hard surface has been observed previously in the oscillations of the shank and attributed to fluctuations in the distal leg during equilibrium maintenance with a non-stiff posture [63]. In a sense, there is greater freedom of oscillating capacity on hard than compliant support, whereas sensory and motor constraints connected to the unsteady condition (foam) are instead prominent and impact on and continuously modify the timing of the leg muscle reflexes [64,65]. The lower and broader spectrum profile on hard support would be linked to periods of stillness, so that the dominant 4 Hz frequency of the VGRF oscillations on foam becomes “less dominant” or less regular because there are longer intervals between body motions (the left tail of the bell curve of the VGRF spectrum). Such a process would underpin an intermittent-feedback control strategy [66,67,68], where the feedback is switched off at irregular intervals when the body’s CoM is safely located near the point of upright equilibrium. When stance becomes unstable on the hard surface, balance corrections by co-contraction of postural muscles would occur (a mode hardly fitting on the compliant surface), the frequency of which may be quite high (the right tail of the bell curve) [40,50,69,70].

The relative inflexibility of the “postural rhythm” generator suggests that central activity prevails over proprioceptive sensory feedback from the postural muscles, which is presumably very different between compliant and hard support [33]. Remarkably, the application of mechanical vibration to ankle muscle tendons (therefore adding an external, strong proprioceptive input) in the unstable foam condition does not affect the postural control mode [71]. Further, during stance, the muscle spindle Ia excitation of the motor neurones is decreased, likely by presynaptic inhibition on hard surface both in bipedal or in less stable unipedal conditions [72,73], thereby reducing reactive instability in the reflex loop [34]. Moreover, others have shown that the soleus H-reflex gain decreases with support surface compliance [74]. The stretch reflex excitability modulations may help focus the “postural rhythm” about its dominant frequency. Moreover, this VGRF rhythm is not altered by vision and adaptation, which influences the spectrum amplitude. It is perhaps neurally and computationally more efficient to rely on a uniform rhythm periodicity and modulate the amplitude of the VGRF.

#### Body Weight Substantially Increases VGRF Oscillation Amplitude on Compliant Support but Minimally Affects VGRF Oscillation Frequency

Body sway increases with body weight, be it connected to carried load or obesity status [75,76,77,78,79,80,81]. In our sample, the amplitude of the VGRF oscillations increased significantly with body weight on compliant compared to hard support (either without or with adaptation). The virtual absence of vertical body acceleration on hard support minimises the VGRF oscillations around the body mass to almost zero for all practical purposes [33]. This is probably why the influence of body weight on the geometric measures of CoP sway has not been consistently found under hard support surface conditions [82,83,84,85]. The inertial mass of a body segment modulates the frequency of its tremor when this is present [86,87,88,89]. Mass reduces tremor frequency according to a sqr (K/I) relationship (where K is stiffness and I inertia) [90]. Hence, the absence of a clear-cut effect of body mass on VGRF frequency when standing on the hard support would be linked to increased stiffness (K) due to muscle co-contraction, more likely to occur on hard than on compliant support [34,86]. This would lessen the relative impact of mass on tremor frequency (a discussion on these opposing factors can be found in [91]. In addition, the decrease in the resonant frequency that accompanies the increase in the mass of a body [36,92,93] and the bending of the knees [94] decrease the frequency of the continuous rhythmic vertical oscillations of the GRF [61]. Notably, the resonance frequencies mentioned in the above papers, calculated independently of the VGRF, are very close to the dominant frequency established with the Gaussian fit and would account for the small differences between foam and solid support. The low determination coefficients of the relationship between VGRF frequency of oscillation and body weight seen in the compliant and hard support conditions (0.4 and 0.1, respectively) also suggest that confounding factors occur.

Overall, it appears that the postural rhythm is set by the resonant frequency of the standing body and modulated by the support conditions. This corroborates the idea that a strong central rhythm generator exists, mildly tuned by mechanics rather than changes in neural action. Moreover, this rhythm would not depend on the highly variable sensory return because the latter should be at least partly function of the CoP oscillations, which have different frequencies than those of the VGRF (see below).

### 4.3. The Different Spectrum Profiles of the Oscillations of VGRF, HGRF, and CoP

The oscillations of the vertical components of the force are not related to the oscillations of the CoP (if there were only the pure vertical component, the CoP would not move at all). It is as if only the low frequencies of the VGRF “pass through” the body’s low-pass filter and affect the excursions of the CoP. The low frequencies of VGRF are relatively underrepresented in the spectrum, but they are the ones that count on the CoP displacement. When one considers the HGRF frequencies (in the antero-posterior, AP, and in the medio-lateral, ML, direction), once again, only the relatively low frequencies of VGRF “pass through” to those of HGRF. It can be implied that the largest share of VGRF is actually represented by the vertical component—the rhythm that controls the effort to maintain the body in equilibrium against gravity—and that when HGRF and CoP shifts occur, this rhythm decreases in frequency.

The angular acceleration of the whole body is due to both the vertical and the horizontal components of the GRF because the direction of the GRFs is not always perpendicular to the support base. Despite the partly overlapping range, the mean spectrum frequencies of the VGRF oscillations are definitely higher than those of HGRF and CoP along both AP and ML directions (Figure 6). The muscles around the ankle (the plantarflexor and dorsiflexor muscles, as well as the invertor and evertor muscles) would be mainly responsible for the ankle torques and VGRF oscillations [95,96], whereas the hip muscles (abductor/adductor), as well as those around the knee and those of the spine, would rather change the body’s angular momentum around the CoM [2,58,97,98,99,100]. Other studies have also shown that standing on a compliant or on a moving support surface enhances reliance on more proximal muscles and produces CoM movements with different dynamics than those imposed by the VGRF [101,102].

The amplitude of the various frequencies distributed in the VGRF and HGRF spectra may not be necessarily associated because the horizontal forces are related not only to the driving rhythm [27], but to events occurring independently of the “postural rhythm” as well. For instance, the trunk movements, although small and most likely slow, may affect the HGRF (thence the CoP) independently of the VGRF. These oscillations could be produced by the action of the muscles of any body parts having their own inertia and moving with a non-null velocity. When standing still, the CoP would have small excursions or “internal” perturbations that would produce a slow body fall before correcting muscle actions ensue. Of note, the dominant frequency of VGRF is not a function of sway amplitude (Sozzi et al., 2022 b) [33], showing that HGRF and CoP oscillations depend on equilibrium strategies “downstream” of the neural centres controlling VGRF, much as occurs when the motor control programs are translated into locomotion synergies [103].

How precisely the generator of the postural rhythm (working at the dominant VGRF frequency of around 4 Hz) can produce HGRF oscillations along both axes of the horizontal support at a much lower frequency—and CoP oscillations at a still lower frequency content (see below)—is a thorny issue [104,105,106]. The frequencies of HGRF may not be identical to those of CoP because they reflect forces and not displacements. A low-pass filtering process must operate to convert the VGRF rhythm into the HGRF oscillations on the support base onto which subjects stand, and ultimately, to the continuous slow motion of the CoP [107], which is the final outcome of the complex activity put in place by the brain through the musculoskeletal plant. Together, these chained events are responsible for the stabilisation of the CoM and its slow oscillations, featuring frequencies that are about half those of the CoP [104,108,109,110].

Numerous studies have shown that the body cannot be equated to a simple inverted pendulum [50,111,112], where a simple relationship would link HGRF and CoP values. The complexity of the balancing movements must contribute to the disparity between the frequency content of the VGRF, HGRF and CoP spectra. The horizontal translational accelerations of the CoP and the angular acceleration of the body as a whole are both passive, depending on body inertia, and active, due to the balance corrective reactions. Possibly, small, frequent force productions such as from a series of closed-by muscle twitches can merge, much as in the production of a fused tetanus [113], and produce smooth, low-frequency corrections and slow body movements and CoP displacements. As a result, the high frequencies of the HGRF spectrum almost vanish in the spectrum of the CoP, while the low frequencies of HGRF correspond to an ample spectrum of CoP.

#### The CoP Frequencies and the Rambling–Trembling Hypothesis

HGRF and CoP oscillations contain, at least in part, equal frequencies, as seen in Figure 10. However, with a median frequency of about 0.3 Hz to 0.5 Hz and with practically no power beyond 2 Hz (less than 2% of the total power), one wonders where the slow CoP oscillations come from. A large part of the HGRF spectral frequencies do not move past the low-pass filter represented by the body, whereas the lowest frequencies of the HGRF spectra are partly shared with those of the CoP oscillation and contribute a direct effect to the CoP excursion pattern.

These facts can be considered in light of the rambling–trembling hypothesis originated from the CoP decomposition analysis [114,115]. The frequency range of the so-called rambling pattern (<0.4 Hz) of CoP trajectory corresponds to where the spectrum of HGRF falls below the profile of the CoP spectrum. Above that frequency, in the trembling range (>0.4 Hz until about 1.5 Hz to 2.0 Hz, for practical purposes) the amplitude of the HGRF spectrum is greater than that of the CoP spectrum, as if trembling had no major effect on CoP excursions [19,115,116]. Therefore, the HGRF excursions with higher frequency and amplitude than the CoP may provide additional insight into the balance control strategies, since the stiffness-modulated frequency components may go undetected using conventional geometrical indexes of sway [69,70]. At about 0.4 Hz, with some variability depending on the sensory condition [20,33], the CoP and the HGRF spectra intersect, dividing the CoP spectra into 2 parts. Remarkably, these points of intersection coincide with the median frequency of the CoP oscillations shown in Sozzi et al. (2022) [33], as if the CoP median frequency would separate rambling from trembling. Below the median frequency value of CoP, the reduction in HGRF spectrum amplitude indicates a decline in the active centrally controlled and proactive engagement of the postural muscles of the standing subjects [19]. Instead, when the CoP spectrum diminishes in amplitude, as occurs above its median frequency value, the orientation of the body would be close to the vertical, controlled by the increased stiffness of the postural muscles [70], believed to be indicative of reactive stability control.

### 4.4. The Cross-Correlation between HGRF and CoP Time Series

The magnitude of the HGRF increases the farther the CoP moves away from its mean position. This is expected because the module of these forces is proportional to the distance between the CoM and the CoP [117,118], and is in opposition of phase [119]. Such a pattern is also normally present when standing on a mobile platform moving in a sinusoidal mode, in which case the phase–opposition pattern is critically modified only when the frequency of the platform motion gradually increases or decreases [120].

Despite their different frequencies, the time series of HGRF and CoP are definitely, even if modestly, correlated. The cross-correlation values are greater with EC than with EO and on foam than on solid (possibly because the amplitude of the CoP oscillations and HGRFs are greater under ECF). The correlation coefficients of the association depend on the relatively small range of the overlapping frequency in the spectra of HGRF and CoP traces, as touched upon above. Further, as indicated by the shift towards low intersection points between the two spectra passing from hard to compliant surface and from EC to EO, sensory conditions affect the strength of the correlation. So, there is a window of frequencies (between 0.3 Hz and 0.5 Hz) in a range where both the CoP and the HGRF spectra show a considerable amplitude. These frequencies would be those contributing mostly to the fair cross-correlation coefficients of the two time-series. These cross-correlation values would be possibly due to the larger amplitude of the CoP oscillations and HGRFs under unstable conditions. On top of that, confounding events would intrude due to undetected body motions. The value of the cross-correlation coefficient decreases with adaptation because the two spectra become almost unrelated, mostly due to the decrease in the CoP spectrum amplitude at high frequency (>0.5 Hz). Therefore, the width of the common frequency range, responsible for the large non-adapted coefficients, becomes shorter, thereby blurring the correlation. This would also explain why, with adaptation, the points at which the two spectra have similar amplitudes (intersection point) move a little towards low frequency values.

### 4.5. The HGRF Module Determines the CoP Position

The CoP displacement away from its mean position corresponds to a large, disproportionate increase in the values of the horizontal forces. When the HGRFs are zero or nearly so, i.e., when the CoP is in its mean position, the ground projection of the CoM would coincide with the CoP [121,122]. When the difference between the CoP position and the CoM projection increases, the CoM acceleration also increases [45,118]. When CoP is ahead of the CoM, the CoM is being accelerated backward and vice versa. Similarly, if the CoP is displaced in the frontal plane compared to the CoM, the latter is accelerated to the left or to the right. The horizontal forces that are in counter-phase to the position of the CoP are the ones that cause the motion of the CoM. By looking at the red skeins of forces in Figure 11, the largest, slow force variations in HGRF are more obvious in the outermost regions of the skein where the CoP oscillations lie further away from its medium position. Hence, the low frequencies prevail around the limits of stability.

The non-perfect coincidence and opposition of phase between HGRF and CoP again illustrates that the rigid pendulum model cannot correspond to the real balancing operation of a standing body, at least under our conditions, particularly on compliant support [21,61,123]. The behaviours of HGRF and CoP are substantially similar between AP and ML. Since HGRF is omnidirectional, the deviations of the body from the vertical are also omnidirectional. Despite minor differences in the median frequency, only moderately larger in AP than ML direction when standing on hard surface, the bowl-shape of the HGRF modules is very similar in the two directions of the supporting surface. This would point to fundamentally similar balance control characteristics, similarly influenced by the complex motion of various body segments. In other words, the differences from the inverted-pendulum mode of control are homogeneous and concordant in all directions of sway. This is true regardless of the characteristics of the support surface, even when the amplitude of the body sway is unquestionably different, and in spite of the feet being not joint. In the compliant surface condition, the AP and ML behaviour of the HGRF and CoP displacement must be affected by the unstable posture creating a complex mode of balancing, favouring a pivoting role of CoM [124,125,126].

### 4.6. The Effects of Adaptation Are Obvious Only in the Most Critical Standing Condition

The brain provides flexible integration of multisensory signals. This is a complex process, particularly critical when sensory input is continuously changing [24]. Signals from diverse receptors and sensory organs must undergo re-weighting in a task-specific manner. Adaptation while standing, with progressive reduction of CoP sway amplitude, would depend on explicit knowledge of the degree of instability [127,128] and on implicit subconscious operation [129]. Under different conditions (arm movement and blurring of proprioception by wrist vibration) adaptation to the mechanical distortion of the movement execution was substantially impaired, suggesting that intact proprioception helps the body find the “adapted” condition [130]. Such process of multi-sensory reweighting [131] would imply learning a complex, successful form of balance organisation. It can evolve on a relatively short-term basis following a rapid sequence of standing trials [8,20,132,133,134,135]. The process of adaptation is dependent on the standing condition at hand (compliant support and absence of vision) but originates from the integrative and learning capacities of the central nervous system as opposed to the sensory input per se [20,136,137,138,139,140]. Further, part at least of the large initial sway of the first trial on compliant surface without vision would be connected to a sensory conflict between proprioceptive and vestibular input and possibly anxiety [128,141], which would gradually subside with the progressive reduction in the H-reflex gain [142].

Adaptation to repeated standing trials hardly modifies the frequency of the VGRF and HGRF oscillations, regardless of vision and support conditions. In other words, even if adaptation markedly reduces the amplitude of their spectra (particularly in the ECF condition), the frequency remains almost unchanged. Hence, the process underpinning the adaptation does not affect/include the frequency modulation of the “postural rhythm”, even if the peak power of the spectrum is reduced for both VGRF and HGRF. Therefore, adaptation would operate by mitigating the energy (or the spectrum amplitude) of the GRFs, and hence, the effort exerted by the responsible muscles, but not the control of timing. Under different conditions (adaptation to repeated postural perturbation), the timing of the balance correcting responses was constant despite large changes in their amplitude [134]. Observed from the present adaptation findings, the inflexibility of the dominant frequency of the VGRF and HGRF represents a strong attractor, constraining the progressive tuning of the standing effort, thereby minimising the oscillation amplitudes of the standing body [143].

Of note, adaptation with eyes closed does not reduce the amplitude of the oscillations to the same level as that observed with vision (see below). Persistence of relatively large oscillations on the compliant surface might prevent the mechanical tactile information from the foot sole or from muscle sensory inflow from fading away [64,144,145,146], thereby allowing uninterrupted skin and proprioceptive input to reach the brain for optimal control [14,147]. Adaptation does instead affect the amplitude and frequency of oscillation of the CoP [10], an effect evident in the CoP path length. The median frequency of the CoP oscillations is reduced with adaptation, hence the reduced path length. The change in the CoP median frequency originates in an increase in the low-frequency range, and a decrease in the high-frequency range normally associated to muscle co-contraction and likely to individual-specific dynamics [110,148]. Co-contraction of postural muscles often occurs at the beginning of a standing task [149] but is not functional to stability [150]. Moreover, co-contraction would be counter-productive when standing on foam because rigidifying the body can be a cause of toppling over [151,152].

#### 4.6.1. Vision

The stabilising effect of vision, already observed in countless studies, was reproduced here [8,61,70,97,101,153,154]. The visual target was the same under compliant and hard support, but a reduction in body sway with eyes open compared to eyes closed was present only with compliant support. Indeed,, the feet-apart position [155,156] probably minimised sway regardless of the visual input on hard support [157]. It has been already shown in an elderly population that visual problems were not associated with body sway when subjects stood on a firm base. However, when standing on a compliant surface, body sway increased with poor visual acuity.

Moreover, vision availability per se should not be considered a condition favouring adaptation under the present conditions [129,138], because no adaptation in the frequency and amplitude of the GRFs (both VGRF and HGRF) and in CoP Path Length were observed with eyes open despite standing on foam. Possibly, vision is ineffective on the process of progressive diminution of the sway amplitudes across subsequent trials just because the oscillations are smaller anyway with than without vision. The selective adaptation in the ECF condition thus seems to be rather related to a threshold of oscillation amplitude necessary for triggering the process, i.e., when oscillations are initially large and standing is difficult, adaptation occurs [158]. With eyes open on compliant support, the oscillations would be larger than with eyes closed on hard support, but not large enough to trigger an adaptation process. The trials on hard support show instead no adaptation even with eyes closed. While standing on compliant support is a new experience for many subjects, standing on hard support would be already “adapted” because it is the “usual” standing condition.

#### 4.6.2. Vestibular Input

Stability in the ECF condition is certainly reliant upon vestibular cues [159,160]. Hence, the eyes-closed task on complaint support likely prioritises vestibular cues [56,161]. When vestibular stimuli are imprecise or not expected as in the most critical standing condition, the vestibulo-spinal reflex pathways generate robust responses that help maintain posture [162,163]. The period of adaptation, with a gradual and more focused vestibular input, would be appropriate to reach an “easy” stationary condition. During the adaptation period one becomes able to decode and exploit the massive proprioceptive information, particularly on the compliant support, and integrate it with the unusual vestibular input [164]. It would be surprising if the proprioceptive information be deleted altogether to only exploit the vestibular one [165]. If proprioceptive input were really a disturbance when standing on foam, one would learn to suppress it over time with open eyes. However, there is no adaptation when vision is available despite the “proprioceptive disturbance”.

Conversely, compliant support would help channel the massive proprioceptive input and integrate it with the vestibular input. It has been shown that, in a different context, visual feedback enhances the information available to the brain when this generates complex, adaptive motor outputs [166,167,168]. The present protocol was not designed to and could not help in addressing which sensory system(s) are responsible for adaptation or whether cortical sensory processes contribute to postural adaptation [169]. It is noticeable, though, that the time course of adaptation to repeated galvanic vestibular stimulation and muscle vibration [170] was similar to that shown here. It seems more than reasonable that the process of adaptation is a facet of the multi-sensory integration and its interaction with intrinsic high-order processes, and that the responsible centre(s), including the spinal networks [171,172], can be accessed by different time-varying sensory inputs [173,174].

#### 4.6.3. Attentional Demands

Having directly experienced standing on a hard and on a compliant support, we would not exclude that our participants standing on foam performed something of a dual-task, paying continuous attention to the information originating in the visual and somatic senses and matching it to their sense of verticality [128,175,176,177,178,179]. Conversely, standing on a hard support was often reported as boring or unremarkable. Of note, when adding a visual oddball paradigm (a commonly used task for cognitive and attention measurement), the elderly displayed an increase in theta activation over the sensorimotor and occipital cortices [180]. When reaction times were measured as an index of the attention devoted to standing in different postures [181], the attentional demand proved to increase more on compliant than on hard support. Without entering a complex digression on that [182,183,184], we would argue that a potential mechanism of adaptation (in the ECF condition) could be explained by the conversion from an initial dual-task to a single task mode of standing (or to a less demanding attentional task), by learning to cope with the novel sensory–perceptual processes perceived during the initial standing trials. Through adaptation, subjects would move from a highly controlled to a more automatic performance [185]. Attention to the postural task would likely be continuous during ECF, thereby leading to postural control and automaticity improvement [186].

### 4.7. Is the VGRF Rhythm Automatic or Voluntary?

The dominant frequency of the VGRF is lower than that of the physiological tremor [88] and of the orthostatic tremor, a progressive disorder often produced by cerebellar dysfunction [187,188,189,190]. None of our young healthy participants certainly had VGRF oscillation frequencies beyond 10 Hz (the reason for the filter selection), which are almost pathognomonic of primary orthostatic tremor [191]. Interestingly, however, the commonality of the present rhythm with the “slow” orthostatic tremor sometimes found in patients with PD [192,193,194] suggests that basal ganglia malfunction does not prevent the operation of a normal 5 Hz generator. Based on this similarity, it may be presumed that the VGRF frequencies may not be traced to abnormal basal ganglia function.

Standing entails voluntary efforts (particularly on the compliant support and without vision) and reflex actions [195,196]. Standing is controlled by multiple brain centres [185,197], based on implicit knowledge of the capacity of the body to correctly produce suitable reactions [198,199,200]. Maintenance of upright stance occurs despite imprecise and poorly anticipated sensory feedback and uncertain knowledge of the effects of the motor action onto the support surface, so that neither reflex responses nor predetermined muscle synergies would explain coping with critical conditions [201,202,203,204]. This requires the brain to comply with the fortuitous modifications of the sensorimotor feedback [205], where modulation of the receiving sensory cortex by the active motor cortex might play a role [206] (Enomoto et al., 2001). Partial functional disruption of selected cortical areas by transcranial stimulation disturbs body balance, clearly showing that the brain is involved in stance control [207,208].

Recording of brain electrocortical activity has shown the theta power (4–7 Hz) of the EEG in the frontal, central and parietal regions of the cortex to increase when a balance task is challenging [209,210,211,212,213,214,215,216]. The dominant frequencies we have observed in the VGRF spectrum when standing on hard and compliant support are comprised within this range of EEG waves, suggesting a consistent relationship between the two rhythms. On the other hand, the beta oscillations of the EEG (frequency > 13 Hz), observed in sensorimotor cortices and basal ganglia, and also taking part in somatosensory processing and motor control [217,218], are not matched by the frequencies observed in VGRF or HGRF or CoP oscillations, pointing to a loose connection with the processes controlling body sway.

### 4.8. The Postural Rhythm May Not Be Specific to Young Participants

Postural tremors have usually been identified based on the oscillations of the CoP as rendered by a force platform [219,220], not by the VGRF oscillations. The VGRF rhythm of a standing subject would intrude upon the CoP motion only when the body verticality is challenged by alcohol intoxication, producing a broad frequency peak around 5 Hz in the CoP spectrum [221,222]. CoP oscillations in 4–6 Hz frequency band have also been observed in patients with phobic postural vertigo [223] and attributed to co-contraction of postural muscles rather than to a sensorimotor disorder.

The fundamental oscillation frequency of the VGRF is basically reproduced in older healthy persons, even if a slight increase in frequency has been reported [32,224] anyhow comprised within the relatively broad frequency range shown here for stance on hard support. On the clinical side, the peak amplitude of the VGRF oscillation frequency in patients with PD should be considered as a potential marker of PD evolution [30]. In a study of upper limb rest tremor in certain postures in Parkinson’s disease, others have found a frequency almost identical to that found here for the VGRF oscillations of young subjects standing on hard surface [225]. It is tempting to assume that the VGRF frequency would be produced by a central generator normally active for controlling upright posture, and that this rhythm would pass onto other body segments when the legs are not held in check by a healthy brain, as occurs in some patients with PD [194,226].

We would not leave unnoticed that a cardinal feature of freezing of gait in PD consists in oscillations of the legs at frequency around 3 Hz to 8 Hz [227]. We speculate that freezing is no more than an involuntary appearance of the postural VGRF rhythm encroaching into locomotion, be it due to anxiety [228], changes in direction [229] or to disease-related problems [230,231]. Freezing would be linked to the simultaneous and conflicting triggering of postural activity in a walking task, when the rhythm generator for standing is exposed to altered excitability of the basal ganglia circuits or of the networks contributing to attention and executive function [232,233].

### 4.9. Limitations

For a better understanding of the mechanisms underpinning the large amplitude changes of the GRFs and their minor frequency modulation, recording of the electromyogram would help, together with an appropriate motion analysis. We have no measurements of head displacement here, vertical or otherwise [234], which would help infer a potential role of the vestibule, especially during the adaptation process in the compliant support condition without vision. Moreover, we used a single foam pad and stance width, but feet distance changes the way sensory information is integrated [58,196,235,236,237]. Accurate measurement of the feet movements in the vertical axis (such as ankle plantar- or dorsiflexion, in synchronous or alternate way) would help understand the role of the compliant surface in the control of the VGRF and of the medio-lateral and antero-posterior sway. We only used one force plate, whereas separate recording of the GRF by two platforms would yield information on the way the postural rhythm is constrained by inter-feet coordination dynamics [236]. We did not manipulate viewing distance, which can affect coordination and control in standing posture [44]. Further, and worthy of note, the results and conclusions cannot be applied directly to ageing populations because the effects of vision and compliant surface on body sway may be different in the elderly from those described here [150,165,238,239,240,241,242,243,244,245], and reduced peripheral sensory function may degrade their control of sway [56,57].

## 5. Conclusions and Future Directions

The frequencies of oscillations of VGRF, HGRF and CoP are quite different, as per the dominant frequency of their spectra (around 4 Hz, 0.8 Hz and <0.4 Hz, respectively). These frequencies hardly change as a function of the experimental conditions, including adaptation, except for the CoP where the frequencies drop to <0.2 Hz when vision is available on the hard support surface. However, the amplitude of the oscillations is affected to a considerable extent for VGRF, HGRF and CoP as a function of the experimental conditions. For the three variables, the amplitudes decrease in parallel in the order ECF > EOF > ECS ≈ EOS. Adaptation is not influent on the amplitudes of the oscillations, except in the ECF condition. Thus, the specific rhythms of the GRFs do not affect the CoP, but it is the modulus of the forces acting on the ground that determines the changes in the geometric measurements and, ultimately in the body sway. Moreover, the discrepancies in the time-series of the HGRF and CoP oscillations (that apply to both the ML and AP directions) indicate a significant distance in the oscillation mode from that dictated by the inverted pendulum model.

The VGRF dominant frequency appears inflexible to the sensory conditions and likely originates in the brain. It is similar to the cortical theta rhythm but different from other postural rhythms of normal subjects and patients. It is found in some patients, notably patients with Parkinson’s disease in some selected cases, and possibly intrudes into the other fundamental rhythm underpinning locomotion. In consideration of the quite stable dominant frequencies of VGRF and of HGRF, the postural control mode seems to be the expression of a robust operation of brain centres able to move the body with changing amplitudes and directions of motion depending on the sensory and support conditions, but within the constraint of a definite “postural rhythm”. Much remains to be done in order to trace the underpinning mechanisms and to understand which brain regions contribute to the VGRF rhythm or where its central generator is located [5]—or even how sensory information modulates the rhythm. The present findings are neutral as to the role of the cerebellar vermis in postural adaptation [130,246,247,248].

In closing, we suggest that a major challenge would be to understand the transition phase between posture and gait, or, in this context, the transition between the postural rhythm and the locomotor rhythm produced by the central pattern generator for walking [249]. After all, it would seem peculiar that the control of the standing posture was functionally separate from that of locomotion, because one normally starts and stops walking at will without falling, or because one often halts along the way for a number of reasons and for longer or shorter periods of time or finally, stumbles and stands up after the trip almost seamlessly. A smooth transition between standing and walking is not a trivial task [250], not to speak of the problems in movement disorder conditions. For instance, gait initiation is often difficult in PD patients [251], as rapid termination of walking can be [252,253,254]. In this light, the steadiness and dependability of the postural rhythm, relatively unaffected by the external state, must represent a safe reference for the nervous system. It seems that the theta rhythm recorded with the EEG in PD patients signals some abnormality during the prodromal phase of freezing. One could interpret the data collected in PD patients during freezing [255] as the intrusion of posture control mechanisms at the invalid time during gait, which creates a conflict between the postural rhythm and the rhythm of walking.

These results shed new insight on current principles of motor control and might have favourable consequences on the design of future studies on postural control in elderly people [69,239,256,257,258] as well as on injury prevention and rehabilitation. Further, whether this information can help early identification of defects in neurodegenerative diseases featuring balance impairment is an open issue [30,259,260]. As a final plain remark, we recommend the use of compliant surface for balance rehabilitation, not least because it entrains a definite adaptation process, contrary to standing on hard support [261,262].

## Figures and Tables

**Table 1:** Gaussian fit parameters (dominant frequency, peak amplitude and SD) plotted EC vs. EO in the non-adapted and adapted trials.

Conditions	Equation	R^2^	Slope Different from Zero	Difference between Slope of Non-Adapted and Adapted Trials
Dominant frequency				
Foam Non-adapted	y = 0.81x + 0.48	0.63	*p* < 0.001	*p* < 0.01
Foam Adapted	y = 0.32x + 2.37	0.42	*p* < 0.001	
Solid Non-adapted	y = 0.64x + 1.65	0.38	*p* < 0.001	*p* = 0.83
Solid Adapted	y = 0.71x + 1.36	0.25	*p* = 0.012	
Peak amplitude				
Foam Non-adapted	y = 2.01x + 0.04	0.4	*p* < 0.001	*p* = 0.47
Foam Adapted	y = 1.55x + 0.02	0.49	*p* < 0.001	
Solid Non-adapted	y = 0.75x + 0.00005	0.37	*p* < 0.01	*p* = 0.21
Solid Adapted	y = 0.36x + 0.001	0.1	*p* = 0.13	
SD of Gaussian fit				
Foam Non-adapted	y = 0.59x + 0.31	0.24	*p* = 0.015	*p* = 0.053
Foam Adapted	y = 0.07x + 0.96	0.018	*p* = 0.53	
Solid Non-adapted	y = 0.03x + 2.6	0.0009	*p* = 0.89	*p* = 0.83
Solid Adapted	y = 0.1x + 2.18	0.01	*p* = 0.59	

**Table 2:** VGRF mean frequency vs. body weight in the non-adapted and adapted trials.

Conditions	Equation	R^2^	Slope Different from Zero	Difference between Slope of Non-Adapted and Adapted Trials	Difference in Slope between Conditions
ECF Non-adapted	y = −0.018x + 4.88	0.39	*p* < 0.01	*p* = 0.53	vs. ECS: *p* = 0.63 vs. EOF: *p* = 0.77 vs. EOS: *p* = 0.79
ECF Adapted	y = −0.01x + 4.56	0.38	*p* < 0.01		vs. ECS: *p* = 0.83 vs. EOF: *p* = 0.86 vs. EOS: *p* = 0.25
ECS Non-adapted	y = −0.009x + 5.32	0.02	*p* = 0.52	*p* = 0.73	vs. ECF: *p* = 0.63 vs. EOF: *p* = 0.54 vs. EOS: *p* = 0.86
ECS Adapted	y = −0.017x + 5.9	0.05	*p* = 0.29		vs. ECF: *p* = 0.83 vs. EOF: *p* = 0.97 vs. EOS: *p* = 0.46
EOF Non-adapted	y = −0.019x + 5.28	0.49	*p* < 0.001	*p* = 0.86	vs. ECF: *p* = 0.77 vs. ECS: *p* = 0.54 vs. EOS: *p* = 0.69
EOF Adapted	y = −0.016x + 5.02	0.12	*p* = 0.09		vs. ECF: *p* = 0.86 vs. ECS: *p* = 0.97 vs. EOS: *p* = 0.61
EOS Non-adapted	y = −0.014x + 5.61	0.04	*p* = 0.35	*p* = 0.48	vs. ECF: *p* = 0.79 vs. ECS: *p* = 0.86 vs. EOF: *p* = 0.69
EOS Adapted	y = −0.026x + 6.6	0.24	*p* < 0.05		vs. ECF: *p* = 0.25 vs. ECS: *p* = 0.46 vs. EOF: *p* = 0.61

**Table 3:** VGRF peak amplitude vs. body weight in the non-adapted and adapted trials.

Conditions	Equation	R^2^	Slope Different from Zero	Difference between Slope of Non-Adapted and Adapted Trials	Difference in Slope between Conditions
ECF Non-adapted	y = 0.0018x − 0.03	0.26	*p* < 0.05	*p* = 0.60	vs. ECS: *p* < 0.05 vs. EOF: *p* = 0.16 vs. EOS: *p* < 0.05
ECF Adapted	y = 0.001x − 0.048	0.41	*p* < 0.001		vs. ECS: *p* < 0.001 vs. EOF: *p* = 0.24 vs. EOS: *p* < 0.001
ECS Non-adapted	y = 0.0001x − 0.005	0.38	*p* < 0.01	*p* = 0.37	vs. ECF: *p* < 0.05 vs. EOF: *p* < 0.001 vs. EOS: *p* = 0.12
ECS Adapted	y = 0.00007x − 0.003	0.26	*p* < 0.05		vs. ECF: *p* < 0.001 vs. EOF: *p* < 0.001 vs. EOS: *p* = 0.81
EOF Non-adapted	y = 0.0008x − 0.03	0.49	*p* < 0.001	*p* = 0.66	vs. ECF: *p* = 0.16 vs. ECS: *p* < 0.001 vs. EOS: *p* < 0.001
EOF Adapted	y = 0.0009x − 0.04	0.57	*p* < 0.001		vs. ECF: *p* = 0.24 vs. ECS: *p* < 0.001 vs. EOS: *p* < 0.001
EOS Non-adapted	y = 0.00004x − 0.0007	0.09	*p* = 0.13	*p* = 0.68	vs. ECF: *p* < 0.05 vs. ECS: *p* = 0.12 vs. EOF: *p* < 0.001
EOS Adapted	y = 0.00006x − 0.002	0.26	*p* < 0.01		vs. ECF: *p* < 0.001 vs. ECS: *p* = 0.81 vs. EOF: *p* < 0.001

**Table 4:** Intersection points (frequency) of CoP and HGRF spectra in all experimental conditions.

Condition	Non-Adapted		Adapted	
	ML	AP	ML	AP
ECF	0.34	0.36	0.24	0.24
ECS	0.26	0.21	0.26	0.16
EOF	0.23	0.19	0.2	0.18
EOS	0.22	0.18	0.19	0.17

**Table 5:** Mean cross-correlation coefficients (±SD) between HGRF and CoP traces in the non-adapted and adapted trials. The strength of the correlation gradually diminishes from the less to the more stable condition. The correlation coefficients are minimally but consistently smaller in the AP than ML direction.

Conditions	Non-Adapted		Adapted	
	ML	AP	ML	AP
ECF	−0.76 ± 0.08	−0.74 ± 0.07	−0.64 ± 0.06	−0.60 ± 0.09
ECS	−0.55 ± 0.14	−0.49 ± 0.15	−0.50 ± 0.14	−0.47 ± 0.13
EOF	−0.59 ± 0.08	−0.54 ± 0.08	−0.52 ± 0.10	−0.51 ± 0.10
EOS	−0.51 ± 0.10	−0.43 ± 0.11	−0.45 ± 0.16	−0.43 ± 0.12

**Table 6:** CoP Path Length vs. HGRF total variation. The regression lines are fitted on the data reported in Figure 11C.

Condition	Equation	R^2^	Slope Different from Zero	Difference Between Slope of Non-Adapted and Adapted Trials	Difference in Slope Between Conditions
ECF Non-adapted	y = 0.705x + 1.50	0.77	*p* < 0.001	*p* = 0.67	vs. ECS: *p* = 0.83 vs. EOF: *p* = 0.17 vs. EOS: *p* = 0.59
ECF Adapted	y = 0.65x + 1.19	0.76	*p* < 0.001		vs. ECS: *p* < 0.05 vs. EOF: *p* = 0.97 vs. EOS: *p* = 0.26
ECS Non-adapted	y = 0.77x + 0.214	0.58	*p* < 0.001	*p* = 0.06	vs. ECF: *p* = 0.83 vs. EOF: *p* = 0.21 vs. EOS: *p* = 0.68
ECS Adapted	y = 1.19x − 0.207	0.71	*p* < 0.001		vs. ECF: *p* < 0.05 vs. EOF: *p* < 0.05 vs. EOS: *p* < 0.01
EOF Non-adapted	y = 1.06x + 0.03	0.64	*p* < 0.001	*p* = 0.09	vs. ECF: *p* = 0.17 vs. ECS: *p* = 0.21 vs. EOS: *p* = 0.51
EOF Adapted	y = 0.66x + 0.55	0.45	*p* < 0.001		vs. ECF: *p* = 0.97 vs. ECS: *p* < 0.05 vs. EOS: *p* = 0.35
EOS Non-adapted	y = 0.88x + 0.036	0.41	*p* < 0.001	*p* = 0.12	vs. ECF: *p* = 0.59 vs. ECS: *p* = 0.68 vs. EOF: *p* = 0.51
EOS Adapted	y = 0.44x + 0.5	0.24	*p* < 0.05		vs. ECF: *p* = 0.26 vs. ECS: *p* < 0.01 vs. EOF: *p* < 0.001

## Data Availability

The data presented in this study are contained in the article.
